# Engineered 5-HT producing gut probiotic improves gastrointestinal motility and behavior disorder

**DOI:** 10.3389/fcimb.2022.1013952

**Published:** 2022-10-20

**Authors:** Bei Li, Min Li, Yanan Luo, Rong Li, Wei Li, Zhi Liu

**Affiliations:** ^1^ Department of Biotechnology, College of Life Science and Technology, Huazhong University of Science and Technology, Wuhan, China; ^2^ Key Laboratory of Molecular Biophysics, Ministry of Education, Wuhan, China

**Keywords:** *E. coli* Nissle 1917, engineered probiotic, 5-HT, gut microbiota, constipation

## Abstract

Slow transit constipation is an intractable constipation with unknown aetiology and uncertain pathogenesis. The gut microbiota maintains a symbiotic relationship with the host and has an impact on host metabolism. Previous studies have reported that some gut microbes have the ability to produce 5-hydroxytryptamine (5-HT), an important neurotransmitter. However, there are scarce data exploiting the effects of gut microbiota-derived 5-HT in constipation-related disease. We genetically engineered the probiotic *Escherichia coli* Nissle 1917 (EcN-5-HT) for synthesizing 5-HT in situ. The ability of EcN-5-HT to secrete 5-HT *in vitro* and *in vivo* was confirmed. Then, we examined the effects of EcN-5-HT on intestinal motility in a loperamide-induced constipation mouse model. After two weeks of EcN-5-HT oral gavage, the constipation-related symptoms were relieved and gastrointestinal motility were enhanced. Meanwhile, administration of EcN-5-HT alleviated the constipation related depressive-like behaviors. We also observed improved microbiota composition during EcN-5-HT treatment. This work suggests that gut microbiota-derived 5-HT might promise a potential therapeutic strategy for constipation and related behavioral disorders.

## Introduction

Chronic constipation (CC) is a common functional gastrointestinal (GI) disorder with a 15% global prevalence (Bharucha and Lacy, 2020). Slow transit constipation (STC), a most common type of chronic constipation in clinical practice, is characterized by markedly increased total bowel transit time (Jamshed et al., 2011). STC can cause abdominal distention, pain, nausea, vomiting, perianal illness, and even colon cancer (Stern and Davis, 2016). The persistent occurrence of STC symptoms cause great distress to the patients and impairs their quality of life. Medical managements for STC include laxatives, motilin receptor agonist, prokinetic and other agents (Tillou and Poylin, 2017). However, the associated side effects, tolerance and dependencies of these drugs highlight the need for novel therapeutics (Ramkumar and Rao, 2005).

5-hydroxytryptamine (serotonin, 5-HT) is an important neurotransmitter and plays crucial roles in regulating host mood, memory, appetite, intestinal homeostasis and metabolism (Berger et al., 2009). The majority of 5-HT (up to 95%) is produced by enterochromaffin (EC) cells in the gut, despite its key roles in the central nervous system. (Gershon and Tack, 2007). Previous studies had proposed that endogenous 5-HT was an important enteric neurotransmitter. However, recent studies have shown that 5-HT antagonists still have the same or greater inhibitory effect on GI-motility and transit, even when all endogenous 5-HT has been genetically (Spencer et al., 2013) or pharmacologically (Yadav et al., 2010) ablated from the gut. However, exogenous 5-HT potently increases GI transit in many species tested  (Spencer and Keating, 2022).

When 5-HT released by EC cells binds to different subtypes of 5-HT receptors (5-HTRs) in the intestinal lumen and lamina propria, a variety of important physiological activities are manipulated. The G-protein-coupled receptor (GPCR) 5-HTR4, which is present in the epithelium of the entire colon, is the most exposed 5-HTR to the lumen (Hoffman et al., 2012a). With its role in promoting motility and intestinal secretion control, 5-HTR4 has been targeted in diseases associated with slow GI transit, such as IBS-C (Cole and Rabasseda, 2004). Prucalopride, a very highly selective 5-HTR4 agonist, is developed as an orally administered, first-in-class drug for treatment of severe chronic constipation (Jiang et al., 2015).

Over recent decades, there has been a growing appreciation of the role of gut microbiota in the maintenance of human health. Recently, several studies have also shed light on the effect of microbiota in gut motility (Chandrasekharan et al., 2019; Obata et al., 2020; Wang et al., 2020). As probiotics can be delivered orally, enhance targeting drug efficacy and minimize systemic side effects, engineering them as therapeutics has garnered increasing interests. Since its discovery in 1917, *Escherichia coli* strain Nissle 1917 (EcN), a commensal bacterium in human gastrointestinal tract, has been successfully used in clinical applications to treat a variety of GI disorders (Henker et al., 2007; Schultz, 2008; Wassenaar, 2016). Owing to its excellent safety profile and genetic tractability, EcN has been modified as a versatile probiotic strain and proven to be effective for treating numerous diseases, including antitumor drug carriers, pathogens resistance, immunotherapy and metabolic abnormalities improvement (Hwang et al., 2017; Ho et al., 2018; Praveschotinunt et al., 2019).

In this study, we genetically inserted the rice (*Oryza sativa*) tryptophan decarboxylase gene *tdc(R)* into the chromosome of EcN (EcN-5-HT) to produce 5-HT. Then, we tested this system *in vivo* using a murine model of constipation. The oral delivered engineered probiotic showed therapeutic activities that efficiently alleviated constipation symptoms and related behaviour disorders. Furthermore, 5-HTR activation and microbiota regulation involved in the underline mechanisms were discussed.

## Materials and methods

### Strains and media

All bacterial strains and plasmid used in this study are listed in **Supplementary Table 1** and **Supplementary Table 2**. EcN (non-pathogenic probiotic isolate, serotype O6: K5: H1) was kindly provided as a gift from Jun Zhu’s lab (University of Pennsylvania).

Luria-Bertani (LB) medium with appropriate antibiotic selection (100 μg ml^−1^ ampicillin,100 μg ml^−1^ kanamycin) was used for cell cultivation. Cell growth was monitored by OD_600_ measurements. Modified M9 medium (M9Y) with 10 mM tryptophan, 1 mM BH4, 0.1% Casein Hydrolysate, 50 µg/mL FeCl3 and 0.2% ZYT (1.6% tryptone, 1% yeast extract, 0.5% NaCl) was used for production of 5-HT in shake flasks. EcN-5-HT was incubated in LB at 37°C overnight. Then, the medium was centrifuged at 8,000×g for 10 min to obtain the supernatant. The production of 5-HT in fermentation supernatant was measured by UPLC-MS/MS.

Full-length of tryptophan decarboxylase (TDC) cDNAs from *Catharanthus roseus* (GenBank accession no. MG748691.1), *Oryza sativa* Japonica Group (GenBank accession no. AK069031) and *Bacillus atrophaeus* strain C89 (GenBank accession no. JQ400024.1) were codon optimized and synthesized by Genewiz (Suzhou, China). Then, flanking gene fragments were cloned into pACYC-araBAD plasmid backbone using NEBuilder HiFi DNA Assembly (NEB, Ipswich, USA) to create pACYC-araBAD-TDCs. Next, pACYC-araBAD-TDC expression systems were transferred to EcN-5-HTP strain using a MicroPulser electroporator (Bio-Rad, CA, USA) following the manufacturer’s instructions. Clones were cultivated on LB agar supplemented with kanamycin (100 μg ml^−1^) at 37°C. Modifications were verified by PCR and gene sequencing (Tsingke, Beijing, China) (**Supplementary Figure 1**).

For genome integration of TDC (R), a λ-Red recombination system was employed (Datsenko and Wanner, 2000). All primers for the integration used in this study are given in **Supplementary Table 3**.

### Animal model

Specific pathogen-free (SPF) C57BL/6J mice (6 weeks old, male) were purchased from the Hubei Province Center for Disease Control and Prevention (Wuhan, China). The mice were housed (no more than four per cage) under humidity- and temperature-controlled conditions and a 12-hour light/dark cycle with free access to food and water. All animal procedures strictly conformed to the Guide for the Care and Use of Laboratory Animals published by the United States National Institutes of Health.

After a week of adaptive feeding, mice were randomly divided into five groups (n=8): a normal group, a model group, a EcN WT group, a EcN-5-HT group and a prucalopride group. The loperamide-induced constipation model was established by 7 days of twice-daily (9:00 and 18:00) intraperitoneal administration (*i.p.*) of loperamide hydrochloride (8 mg/kg body weight, 200 μL) suspended in physiological saline in all the groups except the normal group. After that, EcN WT/EcN-5-HT (1×10^9^ CFU suspended in 100 μL of saline) was given through gavage every two days to the mice for 14 days. For the prucalopride group, 2 mg/kg body weight prucalopride was oral administered daily to the mice through gavage (Zhang et al., 2018), while mice from the normal group were treated with saline. Mice were sacrificed and samples were collected at the end of the treatment period.

### GI motility

Stool water content was calculated as [(initial stool weight − dry stool weight)/initial stool weight] × 100%. Stool frequency was measured as the number of stool pellets extruded from each mice per hour. For defecation time measurement, mice were oral administrated of 10% activated carbon, and were given free access to food and water, the time between the gavage and the appearance of their first darkened feces was recorded (Liu et al., 2020). At the end of the experiment, each mouse was gavaged with 0.2 ml of activated carbon solution and sacrificed after 30 minutes. The GI transit time was determined by recording the length of the small intestine and the distance traveled by the activated carbon in the intestine.

### UPLC–MS/MS

Bacterial supernatant or tissue homogenate was extracted with 70% methanol, vortexed and centrifuged at 14000×g for 15 min at 4°C. 5-HT was separated and detected on an AB Sciex 4500 UPLC-MS/MS system (AB Sciex, USA). Samples were injected (2 μl) and separated on a Waters BEH C18 column (Water, USA) (100mm×2.1mm×1.7μm). The mobile phase consisted of solution A (5mM ammonium formate, 0.1% formic acid) and solution B (0.1% formic acid in acetonitrile) at 0.3 ml min^−1^ at 40°C. The gradient elution was programed as follows: 0-1 min, 95% A; 1-2 min, 95-10% A; 2-4.5 min; 10% A; 4.5-4.6 min; 10-95% A and 4.6-7 min, 95% A. 5-HT was detected using selected reaction monitoring of compound-specific mass transitions in positive electrospray ionization mode: m/z 177 > 160 for the qualitative ion pair of 5-HT; m/z 177 > 132.1, 177 > 115.1 for the quantitative ion pair of 5-HT. Data acquisition and processing were performed with the analyst software MultiQuant 2.1.1.

### Histological analysis

For immunofluorescence staining of 5-HT and CgA, frozen slices of the dissected colon tissues from different groups were blocked with 5% BSA in PBS for 60 min. Heat mediated antigen retrieval was performed in 0.01M citrate-buffer (pH 6.0). The slices were then incubated with a 1:500 dilution of anti-serotonin antibody (ab6336, Abcam, USA) and anti-Chromogranin A antibody (ab283265, Abcam, USA) rocked on an orbital shaker (Mini Roller, NEST Biotechnology, China) at 4°C in the dark overnight. Afterwards the slices were treated with HRP-conjugated secondary antibody, in PBS at room temperature in the dark for 60 min. Cell nuclei were stained with DAPI (Sigma, USA). Stained cells were then visualized by fluorescence microscopy (Nikon Eclipse CI, Japan).

### Gene expression

RNA from harvested colonic tissues was extracted with TRIzol reagent (Invitrogen, USA). To generate cDNA, we used the HiScript II 1st Strand cDNA Synthesis Kit (Vazyme, China) with 2 μg of RNA for each sample. mRNA relative expression was measured using a CFX Connect Real-Time PCR Detection System (Bio-Rad). PCR was carried out with 10 μL of SYBR Green Master Mix (Yeasen, Shanghai, China), 2 μL of complementary DNA (cDNA), 0.4 μL of forward primer, 0.4 μL of reverse primer, and 7.2 μL of nuclease-free water. The samples were subjected to 40 cycles of amplification. Preincubation was for 30 seconds at 95°C, followed by denaturation at 95°C for 10 seconds, annealing at 58°C for 20 seconds, and extension at 72°C for 30 seconds. The primers used in the present study are listed in **Supplementary Table 4**.

### Behavior test

Open field test (OFT): Briefly, mice were gently placed in an open field, a white plastic box (46×46×40 cm). The center was located in 3/5 places of length and width. Mice were placed in the center of the arena tracked for 10 min. Elevated plus maze test (EPMT): Mice were placed in the center part of the maze facing one of the two open arms. Mice behavior was tracked for 10 min. Forced swim test (FST): Mice were gently placed in transparent cylindrical tanks (30 cm height×20 cm diameters) containing water (23°C ± 2°C) with 15 cm in depth from the bottom. After 2 minutes for acclimation, the immobility time was recorded for 6 minutes. Tail suspension test (TST): Mice were suspended upside down by tails 40 cm above the floor by adhesive tape placed 1 cm from the tail tip and tracked for 6 minutes.

Before the behavioral tests, all mice were allowed to acclimate to the test room for at least 2 hours prior to starting the test. Movements of the subject mice were recorded and analyzed by SMART 3.0 video tracking software (Panlab Harvard, MA, USA).

### Microbial DNA extraction and sequencing

At the end point of treatment, mice fecal samples were collected and frozen at -80°C immediately after collection. Total genomic DNA from approximately 200 mg of stool was extracted by a QIAamp DNA Stool Mini Kit (Qiagen, Valencia, CA) according to the manufacturer’s instructions. The V3-V4 region of the bacterial 16S ribosomal RNA (rRNA) genes was amplified by PCR with universal primers (338F, ACTCCTACGGGAGGCAGCAG; 806R, GGACTACHVGGGTWTCTAAT) and FastPfu Polymerase. Amplicons were then purified by gel extraction (AxyPrep DNA Gel Extraction Kit, Axygen Biosciences, USA) and quantified using QuantiFluor-ST (Promega, USA). The purified amplicons were pooled in equimolar concentrations, and paired-end sequencing was performed using an Illumina MiSeq platform (Illumina, San Diego, USA).

### Statistical analysis

Statistical analysis was performed with GraphPad Prism 8 statistical software. Comparisons between two groups were performed using unpaired two-tailed Student’s t-test. One-way analysis of variance was used for comparisons of more than two groups. The results are presented as the mean ± SD. Differences were considered significant at *P < 0.05, **P < 0.01, ***P < 0.001, and ****P < 0.0001.

## Results

### Construction of 5-HT biosynthetic pathway in EcN

**Figure 1:**
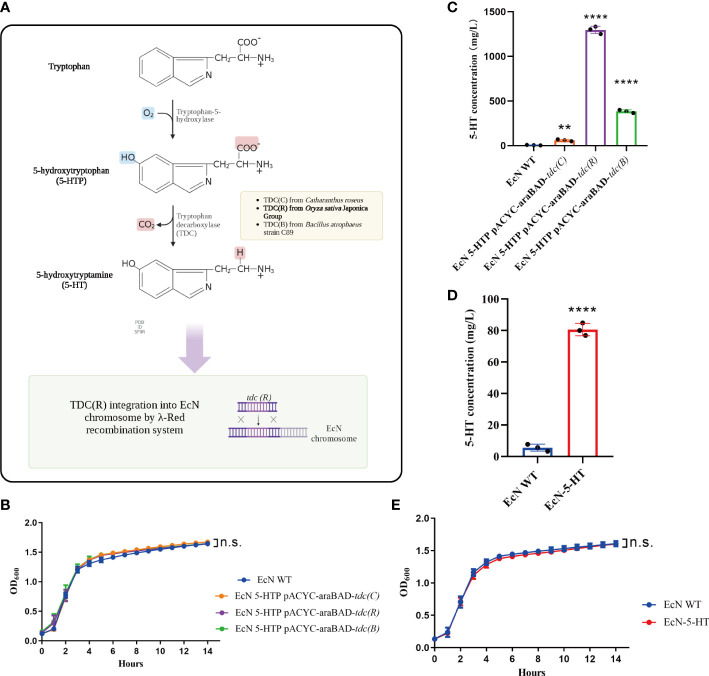
Engineer *Escherichia coli* Nissle 1917 (EcN) to synthesis of human neurotransmitter 5-HT. **(A)** Schematic summarizing the design strategy to engineer EcN-5-HT. **(B)** Growth curve of the three engineered EcN in LB medium. **(C, D)** Measurement of 5-HT production by UPLC-MS/MS. **(E)** Growth curve of the engineered EcN-5-HT in LB medium. Mean values ± SDs are presented, p values were calculated using unpaired t-test, **p < 0.01, ****p < 0.0001, n.s: not significant. Data are pooled from three independent experiments with n = 3 per group.

5-HT is natively produced from 5-HTP by tryptophan decarboxylase in animals and plants. The 5-HT biosynthetic pathway was introduced into a 5-HTP-producing EcN (**Figure 1A**, **Supplementary Figure 2**). To verify the decarboxylase activities and obtain desired products, we tested three tryptophan decarboxylases (TDCs) from *Catharanthus roseus*, *Oryza sativa* Japonica Group, and *Bacillus atrophaeus* strain C89. EcN was transformed with the protein expression plasmid pACYC-araBAD containing the genes encoding TDC under the inducible promoter (P_BAD_). We first confirmed that the introduction of each *tdc* gene didn’t influence the growth of EcN, comparing with the empty plasmid control (**Figure 1B**). Among them, EcN with pACYC-araBAD-*tdc(R)* showed the highest TDC protein yield (**Supplementary Figure 3**). The cell-free supernatants of the three transformed strains were collected separately and detected by UPLC-MS/MS. Results showed that all the supernatants contain 5-HT (**Figure 1C**). As expected, EcN with pACYC-araBAD-*tdc(R)* yield the highest 5-HT level (**Figure 1B**). Then, pACYC-araBAD*-tdc(R)* fragment was integrated into *malEK*, the intergenic region between *malE* and *malK* genes (Kurtz et al., 2019), of EcN using the λ-Red recombination system to ensure the stable expression *in vivo*. The recombinant strain (EcN-5-HT) generated higher production than the control strain (80.6 mg/L *vs*. 5.6 mg/L, **Figure 1D**), and performed a similar growth pattern as the wildtype strain (**Figure 1E**). These results together indicated that EcN-5-HT strain could efficiently secrete 5-HT to the extracellular culture without affecting its growth.

### Gastrointestinal motility enhancement from engineered EcN

**Figure 2:**
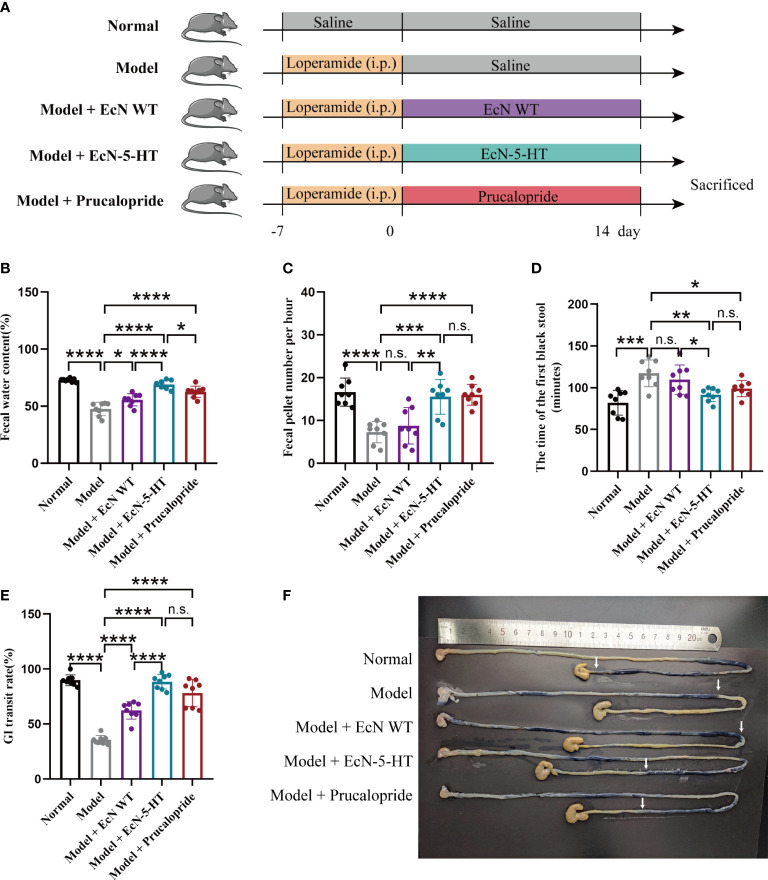
EcN-5-HT improved gastrointestinal motility in a loperamide‐induced constipation model. **(A)** Experimental setup. **(B)** Fecal water relative content. **(C)** Fecal pellet number per hour. **(D)** Time to first black stool defecation. **(E)** GI transit. **(F)** Representative images of small intestine after treatment with activated carbon by gavage. Mean values ± SDs are presented, p values were calculated using unpaired t-test, *p < 0.05, **p < 0.01, ***p < 0.001 and ****p < 0.0001 n.s, not significant. Data are pooled from three independent experiments with n = 8 mice per group.

The roles of EcN-derived 5-HT in the gut were further evaluated in a constipation animal model. Constipation was induced by loperamide in six-week-old male C57BL/6 mice. Then, the mice were orally gavaged with the strain EcN-5-HT for two weeks (**Figure 2A**). The level of EcN in the fecal of treated mice were significantly increased at the endpoint of the experiment (**Supplementary Figure 4**). We observed that mice receiving EcN-5-HT exhibited improved gastrointestinal motility, as evident from an increase in stool water relative content (**Figure 2B**) and frequency of fecal defecations (**Figure 2C**). Notably, EcN-5-HT administration showed a more potent effect on increasing stool water content than prucalopride (**Figure 2B**). Time of the first black stool defecation following the administration of activated carbon is another indicator of the intestinal patency and peristalsis. We found that the time to first black stool defecation was significantly reduced in the EcN-5-HT treated group (**Figure 2D**). Meanwhile, reduction in whole gut transit time was observed after EcN-5-HT administration (**Figures 2E, F**). Besides, the body weight of mice was also monitored. At the endpoint, no acute body weight drop was observed from the above treatments throughout experiment (**Supplementary Figure 5**). Together, our results suggest that administration of EcN-5-HT reverses loperamide-induced disorders in intestinal motility.

### EcN-5-HT administration increases 5-HT accumulation and 5-HT receptors expression in mice

**Figure 3:**
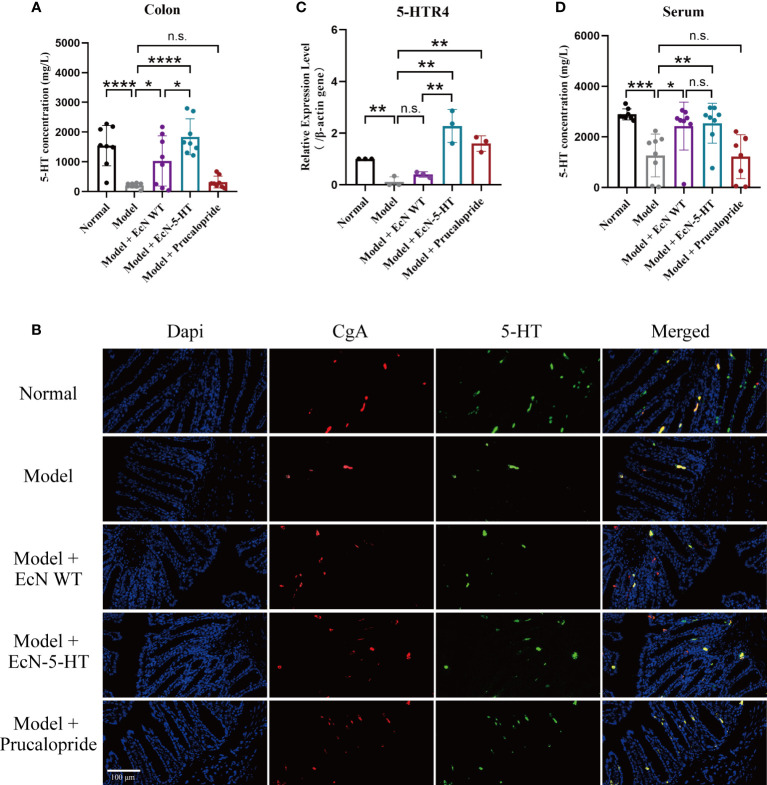
EcN-5-HT led to an increase of 5-HT concentration in constipation mice model. **(A)** Measurement of colon 5-HT by UPLC-MS/MS. n = 8 mice per group **(B)** Fluorescent microscope pictures of colon showing CgA antibody staining (red), 5-HT antibody staining (green) and cell nuclei (blue). **(C)** 5-HTR4 mRNA expression in colon tissue. n = 3 mice per group. **(D)** Measurement of serum 5-HT by LC-MS. n = 8 mice per group. Mean values ± SDs are presented, p values were calculated using unpaired t-test, *p < 0.05, **p < 0.01, ***p < 0.001 and ****p < 0.0001. n.s, not significant.

To further explore the mechanisms of EcN-5-HT strain in regulation of GI motility, we first detected the concentration of 5-HT *in vivo*. UPLC-MS/MS measurements revealed that engineered EcN treatment led to a significant increase of 5-HT yield in mice colon (**Figure 3A**). Colon tissue samples were further processed for immunofluorescence assay and confirmed that content of 5-HT in colon was increased by EcN-5-HT administration, whereas enteroendocrine cells identified by anti-chromogranin A showed no significant group differences in variances (**Figure 3B**). 5-HTR4 is an important therapeutic target for treatment of chronic constipation (Hoffman et al., 2012b; Gwynne and Bornstein, 2019). After different modalities of treatment in healthy or gastrointestinal function disturbed rodent models, the secretion of 5-HT increases, and the expression of 5-HTR4 receptor is upregulated, suggesting that 5-HT and 5-HTR4 receptors may be correlated (Orlando et al., 2020; Yaghoubfar et al., 2020; Zhu et al., 2020). Given the effect of EcN-5-HT on the 5-HT level in colon, we investigated the expression of 5-HTR4 gene. The results showed that expression of 5-HTR4 gene was significantly increased in EcN-5-HT group (**Figure 3C**). On the other hand, the concentration of 5-HT in the serum showed no significant increase in EcN-5-HT group, suggesting that the effect of EcN-5-HT is more significant locally in the intestine (**Figure 3D**). Collectively, the elevated level of 5-HT and upregulated 5-HT receptors in EcN-5-HT treatment group leads to positive effects on intestinal motility.

### Amelioration of depression-like behaviors in loperamide induced constipation mouse model

**Figure 4:**
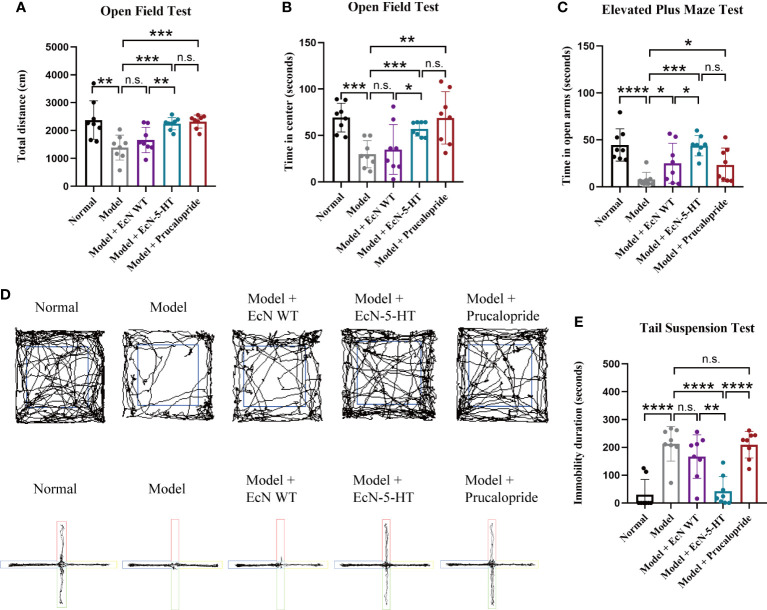
EcN-5-HT ameliorated loperamide-induced behavior disorders. **(A)** Open field test (OFT). **(B)** Representative tracking plots of the open field. **(C)** Elevated plus maze test (EPMT). **(D)** Representative tracking plots of the elevated plus maze test. **(E)** Tail suspension test (TST). Mean values ± SDs are presented, p values were calculated using unpaired t-test. *p < 0.05, **p < 0.01, ***p < 0.001 and ****p < 0.0001, n.s.: not significant.

It has been reported that loperamide-treated mice exhibited significant depressive symptoms (Xu et al., 2018). Therefore, we also tested the behavioral parameters to evaluate the potential role of EcN-5-HT on depressive-like behaviour. Open field test, elevated plus maze test, tail suspension test, and forced swim test are widely used for assessing anxiety-like behaviors and cognitive function. As shown in **Figures 4A, B**, loperamide-treated mice exhibited significantly reduced movement and spent significantly less time in the central region of the open field compared to normal mice. Besides, the model group spent notable less time in the open arms in the EPMT (**Figures 4C, D**) and showed a significantly increased immobility time in the TST (**Figure 4E**). No significant difference was observed between the model and normal groups in FST (**Supplementary Figure 6**). The administration of EcN-5-HT modulated locomotor activity in the OFT and restored the mobility of loperamide-treated mice to control levels (**Figures 4A, B**). On the EPMT, animals in EcN-5-HT group spent significantly more time in the open arms than saline and EcN WT-fed model animals (**Figures 4C, D**). Additionally, EcN-5-HT treatment led to decreased immobile time in TST compared to control mice (**Figure 4E**). Notably, EcN-5-HT showed a better anti-depression effect than prucalopride in TST, suggesting possibly different underlying mechanisms between them. These results indicate that EcN 5-HT ameliorated depression-like behaviors induced by loperamide in mice, suggesting that microbe derived 5-HT can perform anxiolytic effects in host gastrointestinal tract.

### Improvement of gut microbiota dysbiosis by microbiota derived 5-HT

**Figure 5:**
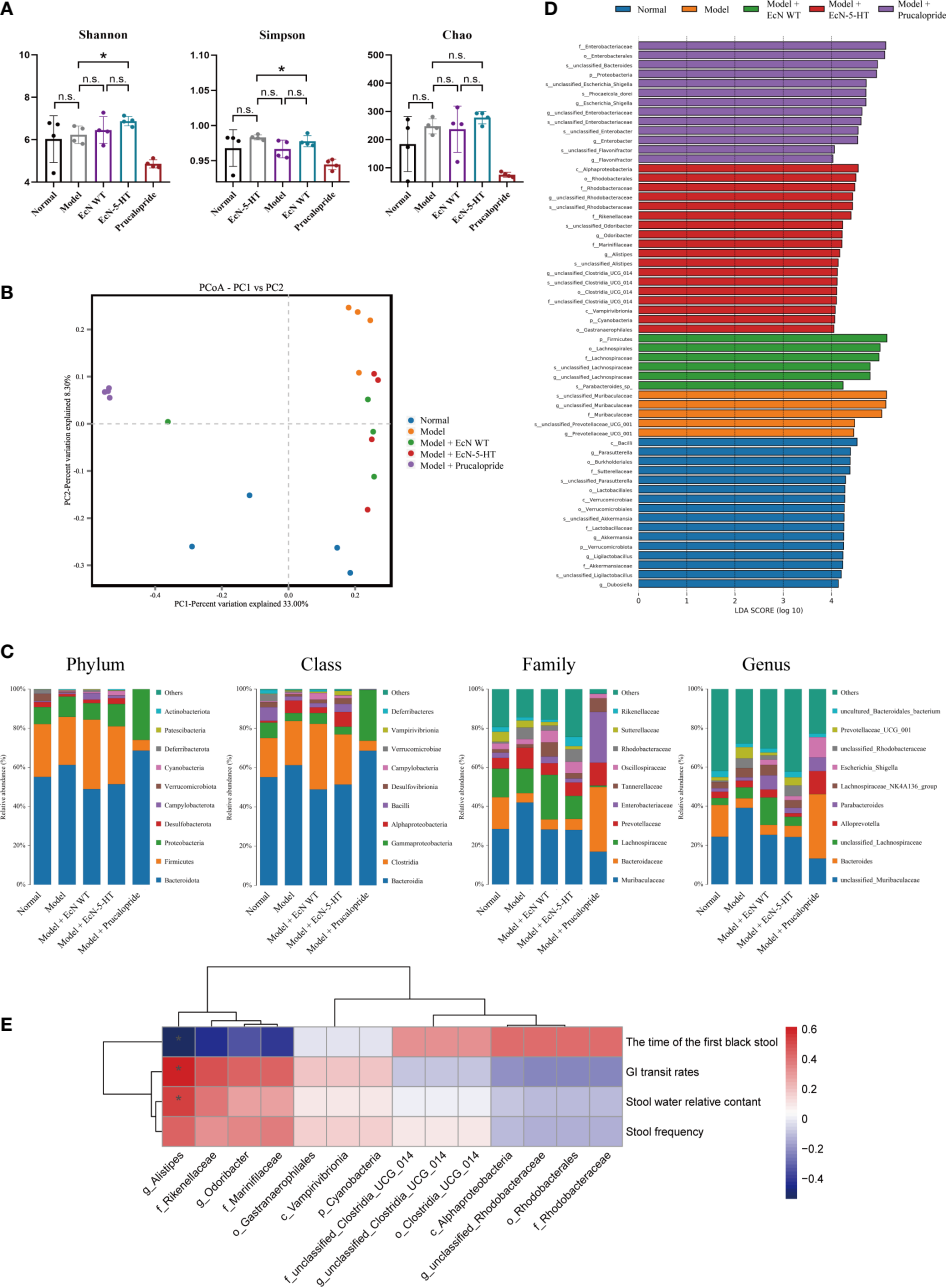
Effects of EcN-5-HT on intestinal microbiota in a constipation mice model. **(A)** Alpha diversity boxplot analysis. **(B)** Principal coordinate analysis (PCoA) profile of microbial diversity. **(C)** Relative abundance of microbial community at different taxonomic levels. **(D)** LDA score computed from features differentially abundant between the groups. **(E)** Spearman correlation analysis. Red and blue colors represent significant positive correlations and negative correlations. The color depth represents the correlation coefficient, and the darker the color, the greater the correlation coefficient. Mean values ± SDs are presented, p values were calculated using unpaired t-test, *p < 0.05, n.s: not significant. Data are pooled from three independent experiments with n = 4 mice per group.

Increasing studies have reported that the microbiota plays important roles in gut motility (Chandrasekharan et al., 2019; Obata et al., 2020). To investigate the influence of microbiota derived 5-HT on gut microbiota composition, we collected the stools from mice at the end of the treatment. Then, microbial DNA extraction and 16S rRNA gene sequencing were conducted. Interestingly, EcN-5-HT treatment significantly increased gut microbiota alpha diversity, including Shannon and Simpson diversity (**Figure 5A**), while the prucalopride treatment resulted in a significant lower alpha diversity (**Figure 5A**). Principal coordinate analysis (PCoA) on OTU levels was also performed to further examine the composition change of gut microbiota between different treatments. The results clearly showed an apparent clustering separation between the normal group and the model group (**Figure 5B**). After EcN-5-HT treatment, the abundance and composition of gut microbiota was more similar to that of the normal group (**Figure 5B**). Classification of OTUs at each phylogenetic level revealed distinct taxonomic patterns between normal mice and constipation mice (**Figure 5C**). To further elucidate the mechanisms of the effect exerted by altered gut microbiota after EcN-5-HT treatment, we performed LEfSe analysis to identify representative abundant bacterial communities among the groups (**Figure 5D**). Results showed that EcN-5-HT treated mice harbored distinctively higher abundances of the genera such as *Alistipes*, *Odoribacter* and *Clostridia* (**Figure 5D**). Relative abundance of *Alistipes* exhibited remarkable and negative correlations with the time of the first black stool and showed significant and positive correlations with GI transit rate, stool water relative content, and stool frequency (**Figure 5E**). Together, these data indicated that EcN-5-HT treatment can improve gut motility by regulating the intestinal microbiota composition.

## Discussion

The role of 5-HT in human health and disease has been widely studied (Lesurtel et al., 2008; Manocha and Khan, 2012; Agus et al., 2018). However, most of the research have focused on host-derived 5-HT. Previous studies have reported that some gut microbes have the ability to produce 5-HT (Özoğul, 2004; Ozogul et al., 2012; O'mahony et al., 2015). The role of gut microbe-derived 5-HT in the gut has not been studied in detail. Although substantial recent evidence has now confirmed that ablation of endogenous 5-HT does not lead to major changes in gastrointestinal transit (Li et al., 2011; Sia et al., 2013; Spencer and Keating, 2022), the findings of the current study imply that synthesis of EcN 5-HT can lead to modifications in GI transit *in vivo*. The mechanisms by which this occurs remains unclear. In our present study, we proved that 5-HT-producing gut microbes can significantly impact gut motility. Our results suggest that microbial 5-HT metabolism could have more implications for GI health, which is barely discussed previously.

Since the 1950’s from work of Bulbring & Crema (Bulbring and Crema, 1959) had provided circumstantial evidence that endogenous 5-HT maybe important in GI motility and transit. However, more recent studies have shown that in fact ablation of endogenous 5-HT has only minor or no effects on GI transit and motility (Spencer and Keating, 2022). Current evidence does not suggest endogenous 5-HT plays a major role, nor is required for control of gut motility or transit *in vivo*. Alterations in the 5-HT pathway are commonly reported in various constipation-related disease conditions. In patients with IBS-C, the content of mucosal 5-HT, the transcription expression of tryptophan hydroxylase 1 transcription and serotonin transporter transcription, and the immunoreactivity of serotonin transporter were all reduced significantly, without any change in the number of enterochromaffin cells (Coates et al., 2004; Wang et al., 2007). In IBS-C patients, postprandial levels of plasma 5-HT were also significantly decreased compared to controls and patients with IBS-D, which may result in significantly delayed gastrointestinal transit (Dunlop et al., 2005; Choi et al., 2014). In colonic inertia patients, lower serotonin receptors in muscular mucosa and circular muscle may contribute to delayed colonic transit (Zhao et al., 2003). In this study, we found no difference in the colonic CgA+ ECs between loperamide-treated mice and normal mice, suggesting that the decreased release of 5-HT by loperamide was not due to the density of ECs (**Figure 3B**).

A number of studies reported a decreased concentration of colon 5-HT in constipation patients, which is consistent with our results (**Figures 3A, B**). Alternatively, several studies also reported higher content of 5-HT in patients with constipation than in normal patients (Lincoln et al., 1990; Costedio et al., 2010). Circulating 5-HT, which represents the 5-HT that is not captured by serotonin transporter (SERT) in the epithelial cells, was used to evaluate the 5-HT availability in the mucosa. More studies on the SERT function in constipation patients are needed in order to guide precise medication of 5-HT-related drugs.

In addition, gut microbiota were involved in 5-HT-related physiology in host. Using antibiotics-depleted microbiota mice model, Ge et al. observed a decreased tryptophan hydroxylase 1 transcriptional expression, 5-HT production, and constipation-like symptoms (Ge et al., 2017). Fecal microbiota from constipation patients led to the same symptoms, including upregulated expression of SERT, and decreased concentration of 5-HT in mice (Cao et al., 2017). These studies suggest that gut microbiota is involved in host 5-HT biosynthesis, and intestinal dysbiosis may contribute to the development of chronic constipation. In this study, by comparing 5-HT producing microbe (EcN-5-HT) with its original strain (EcN WT), we show that gut microbiota-derived 5-HT could improve 5-HTR expression and ameliorated constipation symptoms (**Figures 2**, **3C**). Meanwhile, we observed that EcN WT itself can also lead to an increase of 5-HT in colon and serum (**Figures 3A, D**). It has been reported that EcN is able to enhance host 5-HT bioavailability in intestinal tissues (Nzakizwanayo et al., 2015). This explanation may account for the increase of 5-HT concentration in EcN WT treated mice treated. As shown in **Figure 3C**, there are no significant differences in relative expression of 5-HTR4 between the model and the EcN WT group. It is possible that the colon concentration of 5-HT needs to be high enough in order to activate the 5-HT receptors. The improved GI motility by EcN WT (**Figure 2E**) suggested an additional mechanism independent of 5-HTR4.

Prucalopride, a highly selective 5-HTR4 agonist, is a first-in-class drug for severe chronic constipation treatment (Jiang et al., 2015). Prucalopride treatment can improve stool frequency and consistency, enhanced colonic transit in chronic constipation patients (Müller-Lissner et al., 2010). However, prucalopride side effects have been also reported, such as abdominal pain and diarrhea (Bassotti et al., 2016). In this paper, we observed that prucalopride treatment significantly reduced microbiota alpha diversity (**Figure 5A**) and disrupted microbiota homeostasis (**Figure 5C**). Our results showed that the effects of EcN-5-HT in relieving constipation symptoms are comparable to that of prucalopride (**Figure 2**), along with a positive regulation on the microbiota composition (**Figure 5**). Microbe-derived 5-HT has better effects than prucalopride in the improvement of depression and anxiety induced by constipation (**Figure 4E**), implying different mechanisms between pharmacologic treatment and microbial-derived 5-HT treatment, which requires further investigation.

## Conclusions

**Figure 6:**
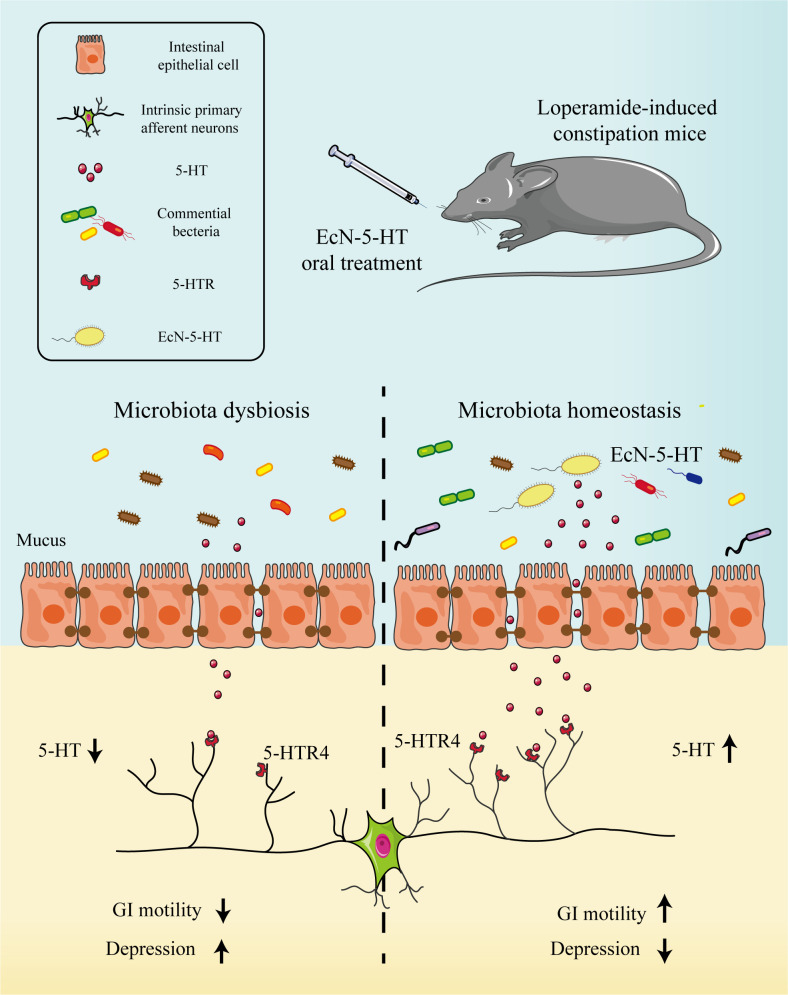
EcN-5-HT improved GI motility and ameliorate behavior disorder in loperamide-induced constipation mice model. EcN-5-HT increased the concentration of 5-HT in colon and activated 5-HT receptors, triggering the peristaltic reflex in the gastrointestinal tract and promoting the GI motility. Meanwhile, EcN-5-HT modified the composition of the intestinal microbiota in loperamide-treated mice.

Although recent studies have confirmed that endogenous 5-HT has a minor role in GI-motility and transit *in vivo*, our data here demonstrate that a genetically engineered probiotic strain (EcN-5-HT) producing 5-HT is able to significantly improve intestinal motility in a murine constipation model (**Figure 6**). EcN-5-HT treatment also greatly improved the gut microbiota homeostasis and significantly relieved depression-like behaviors. Our results suggested that engineered 5-HT producing microbe maybe a promising alternative to the treatment of constipation and related behavior disorders.

## Data availability statement

The datasets presented in this study can be found in online repositories. The names of the repository/repositories and accession number(s) can be found below: https://bigd.big.ac.cn/gsa/browse/CRA007693,PRJNA448831.

## Ethics statement

The animal study was reviewed and approved by Animal Care Committee of Hubei Province.

## Author contributions

ZL, WL, and BL participated in the study design; BL and YL performed the experiments and wrote the manuscript; ML and RL contributed to data analysis and figure drawing. All authors read and approved the final manuscript.

## Funding

This work was supported by the National Key Research and Development Project of China (2019YFA0905600).

## Acknowledgments

The authors gratefully acknowledge helps in the preparation and revision of the manuscript from all members in Liu lab. We thank Research Core Facilities for Life Science (RCFLS) in Huazhong University of Science and Technology for assistance with UPLC-MS/MS analysis.

## Conflict of interest

The authors declare that the research was conducted in the absence of any commercial or financial relationships that could be construed as a potential conflict of interest.

## Publisher’s note

All claims expressed in this article are solely those of the authors and do not necessarily represent those of their affiliated organizations, or those of the publisher, the editors and the reviewers. Any product that may be evaluated in this article, or claim that may be made by its manufacturer, is not guaranteed or endorsed by the publisher.
