# Supplementary material for: Engineered 5-HT producing gut probiotic improves gastrointestinal motility and behavior disorder
Source: Front Cell Infect Microbiol. 2022 Oct 20;12:1013952. doi: 10.3389/fcimb.2022.1013952 (PMC9630942; doi:10.3389/fcimb.2022.1013952)
Supplement: Supplementary file 1 [file DataSheet_1.docx]

Supplementary Material

# Supplementary Figures and Tables

## Supplementary Figures

**
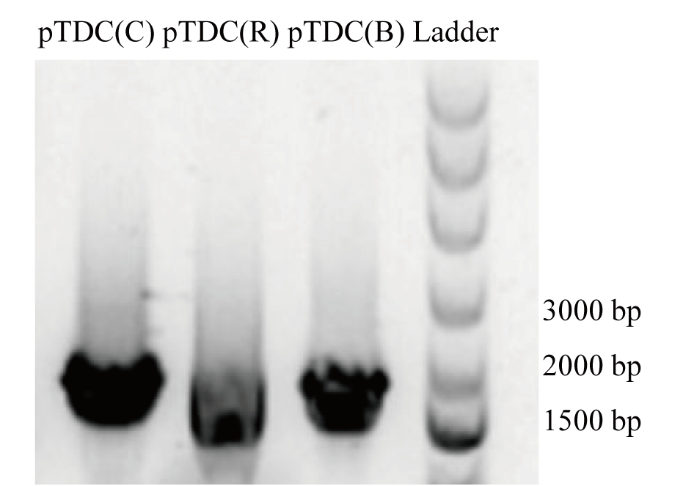
**

**Supplementary Figure 1.** PCR products on agarose gel.


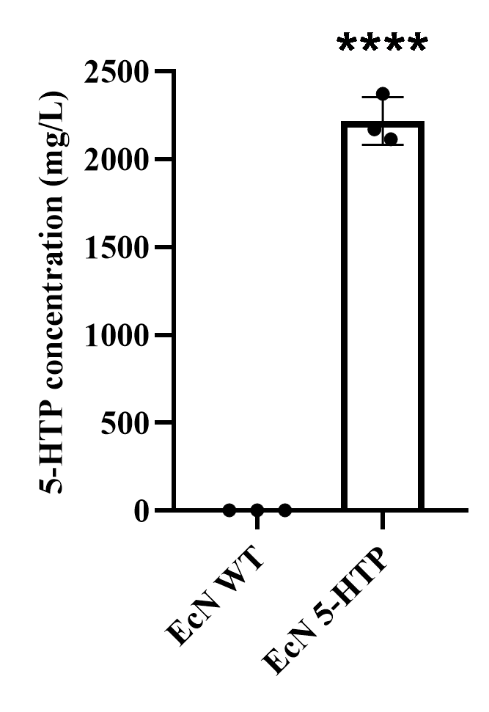


**Supplementary Figure 2.** 5-HTP production of EcN 5-HTP.


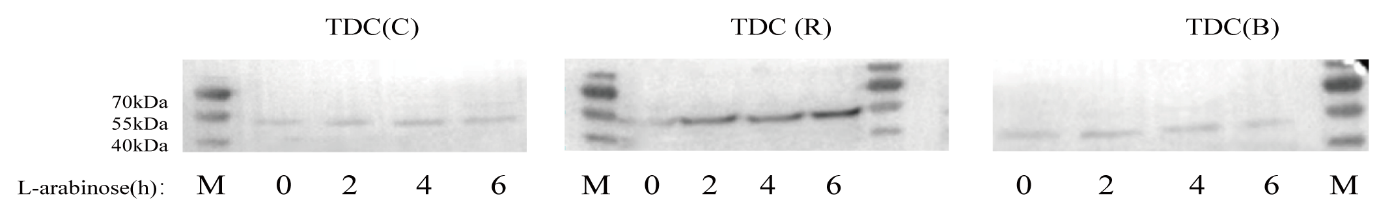
**Supplementary Figure 3.** Protein expression induced by L-arabinose of three TDCs in EcN.


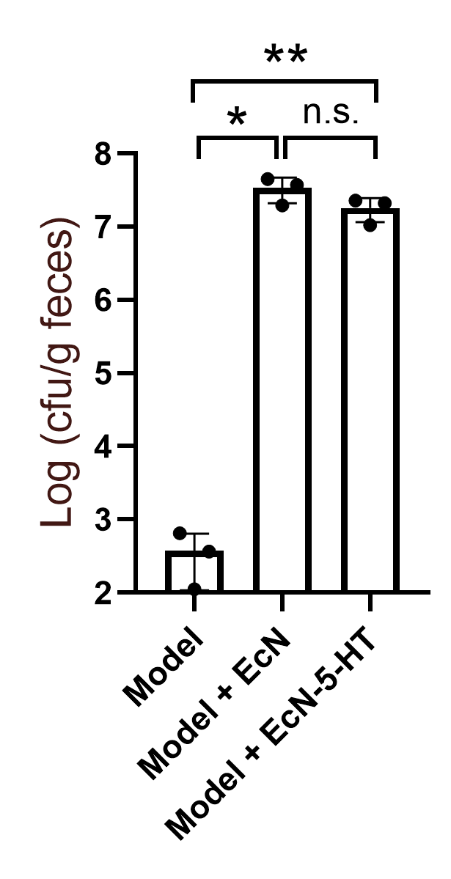


**Supplementary Figure 4.** Real-time PCR with primers specific to EcN. n = 5 mice per group. Mean values ± SD are presented, p values were calculated using Unpaired T-test.

**
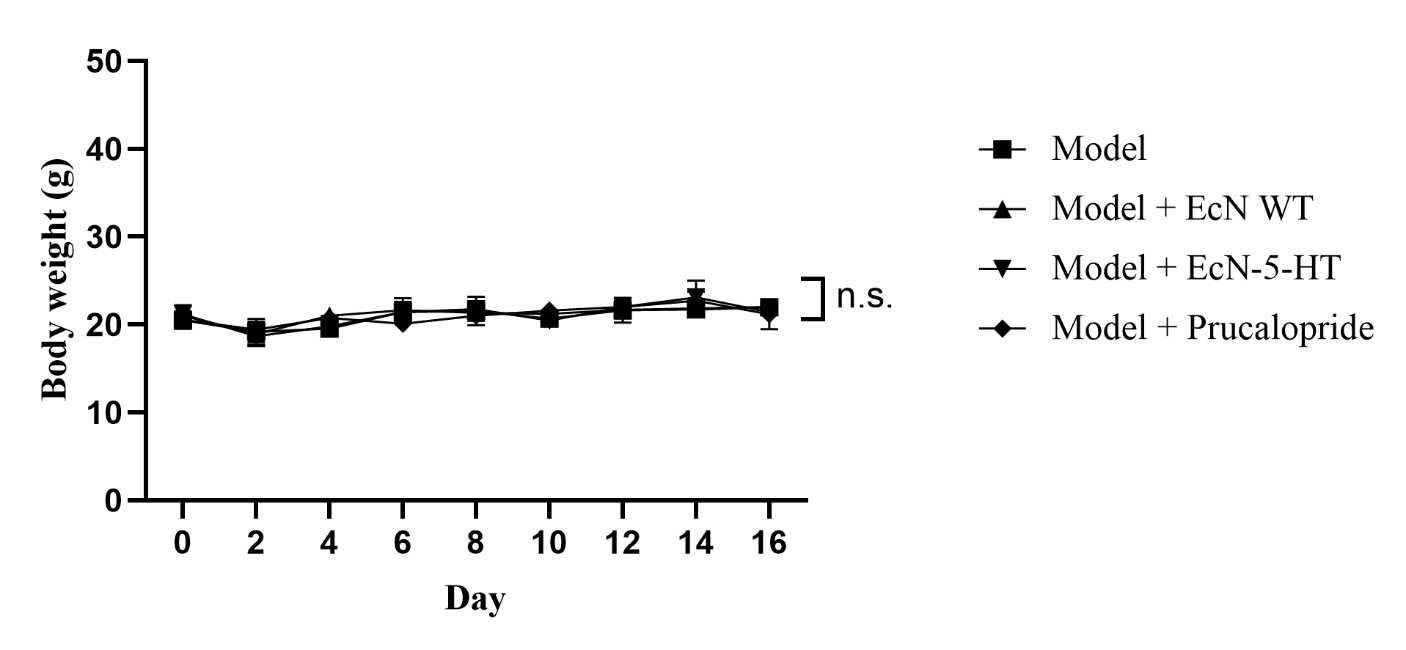
**

**Supplementary Figure 5.** Body weight change. n = 5 mice per group. Mean values ± SD are presented, p values were calculated using Unpaired T-test.


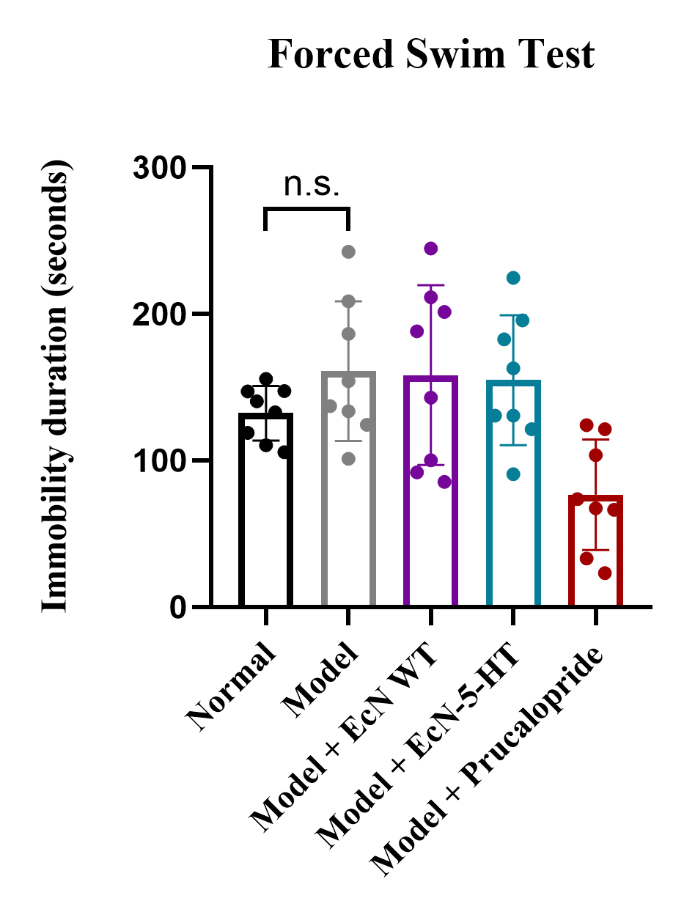


**Supplementary Figure 6.** Immobility duration time in forced swim test.

## Supplementary Tables

**Supplementary Table 1** Strain list.

| Strain | Genotype | Source |
| --- | --- | --- |
| EcN WT | Human commensal E. coli isolate | University of Pennsylvania |
| EcN 5-HTP | EcN Δ*tanA* *lacZ*::hTPH1 | Lab |
| EcN-5-HT | EcN Δ*tanA lacZ*::hTPH1 *malEK*::TDC(R) | This study |
| EcN pACYC-araBAD | EcN with an arabinose inducible plasmid, kanamycin resistance | This study |
| EcN 5-HTP pACYC-araBAD-*tdc(C)* | EcN 5-HTP with an arabinose inducible plasmid expressing TDC(C), kanamycin resistance | This study |
| EcN 5-HTP pACYC-araBAD-*tdc(R)* | EcN 5-HTP with an arabinose inducible plasmid expressing TDC(R), kanamycin resistance | This study |
| EcN 5-HTP pACYC-araBAD-*tdc(B)* | EcN 5-HTP with an arabinose inducible plasmid expressing TDC(B), kanamycin resistance | This study |
| DH5α pKD46 | DH5α with a gene knockout plasmid for the Red system of Escherichia coli, ampicillin resistance | Lab |
| DH5α pKD3 | DH5α with a gene knockout plasmid for the Red system of Escherichia coli, ampicillin resistance | Lab |
| DH5α pCP20 | DH5α with a temperature-sensitive and FLP thermal inducible plasmid, ampicillin and chloramphenicol resistance | Lab |

**Supplementary Table 2** Plasmid sequence.

| Plasmid name | DNA Sequence |
| --- | --- |
| pACYC-araBAD | GTTGACGATGCATAATGTGCCTGTCAAATGGACGAAGCAGGGATTCTGCAAACCCTATGCTACTCCGTCAAGCCGTCAATTGTCTGATTCGTTACCAATTATGACAACTTGACGGCTACATCATTCACTTTTTCTTCACAACCGGCACGGAACTCGCTCGGGCTGGCCCCGGTGCATTTTTTAAATACCCGCGAGAAATAGAGTTGATCGTCAAAACCAACATTGCGACCGACGGTGGCGATAGGCATCCGGGTGGTGCTCAAAAGCAGCTTCGCCTGGCTGATACGTTGGTCCTCGCGCCAGCTTAAGACGCTAATCCCTAACTGCTGGCGGAAAAGATGTGACAGACGCGACGGCGACAAGCAAACATGCTGTGCGACGCTGGCGATATCAAAATTGCTGTCTGCCAGGTGATCGCTGATGTACTGACAAGCCTCGCGTACCCGATTATCCATCGGTGGATGGAGCGACTCGTTAATCGCTTCCATGCGCCGCAGTAACAATTGCTCAAGCAGATTTATCGCCAGCAGCTCCGAATAGCGCCCTTCCCCTTGCCCGGCGTTAATGATTTGCCCAAACAGGTCGCTGAAATGCGGCTGGTGCGCTTCATCCGGGCGAAAGAACCCCGTATTGGCAAATATTGACGGCCAGTTAAGCCATTCATGCCAGTAGGCGCGCGGACGAAAGTAAACCCACTGGTGATACCATTCGCGAGCCTCCGGATGACGACCGTAGTGATGAATCTCTCCTGGCGGGAACAGCAAAATATCACCCGGTCGGCAAACAAATTCTCGTCCCTGATTTTTCACCACCCCCTGACCGCGAATGGTGAGATTGAGAATATAACCTTTCATTCCCAGCGGTCGGTCGATAAAAAAATCGAGATAACCGTTGGCCTCAATCGGCGTTAAACCCGCCACCAGATGGGCATTAAACGAGTATCCCGGCAGCAGGGGATCATTTTGCGCTTCAGCCATACTTTTCATACTCCCGCCATTCAGAGAAGAAACCAATTGTCCATATTGCATCAGACATTGCCGTCACTGCGTCTTTTACTGGCTCTTCTCGCTAACCAAACCGGTAACCCCGCTTATTAAAAGCATTCTGTAACAAAGCGGGACCAAAGCCATGACAAAAACGCGTAACAAAAGTGTCTATAATCACGGCAGAAAAGTCCACATTGATTATTTGCACGGCGTCACACTTTGCTATGCCATAGCATTTTTATCCATAAGATTAGCGGATCCTACCTGACGCTTTTTATCGCAACTCTCTACTGTTTCTCCATACCCGTTTTTTTGGGCTAGCAGGAGGAATTCACCATGGCAATGGCAACAACGTTGCGCAAACTATTAACTGGCGAACTACTTACTCTAGCTTCCCGGCAACAATTAATAGACTGGATGGAGGCGGATAAAGTTGCAGGACCACTTCTGCGCTCGGCCCTTCCGGCTGGCTGGTTTATTGCTGATAAATCTGGAGCCGGTGAGCGTGGGTCTCGCGGTATCATTGCAGCACTGGGGCCAGATGGTAAGCCCTCCCGTATCGTAGTTATCTACACGACGGGGAGTCAGGCAACTATGGATGAACGAAATAGACAGATCGCTGAGATAGGTGCCTCACTGATTAAGCATTGGTAACTGTCAGACCAAGTTTACTCATATATACTTTAGATTGATTTAAAACTTCATTTTTAATTTAAAAGGATCTAGGTGAAGATCCTTTTTGATAATCTCATGACCAAAATCCCTTAACGTGAGTTTTCGTTCCACTGAGCGTCAGACCCCTTAATAAGATGATCTTCTTGAGATCGTTTTGGTCTGCGCGTAATCTCTTGCTCTGAAAACGAAAAAACCGCCTTGCAGGGCGGTTTTTCGAAGGTTCTCTGAGCTACCAACTCTTTGAACCGAGGTAACTGGCTTGGAGGAGCGCAGTCACCAAAACTTGTCCTTTCAGTTTAGCCTTAACCGGCGCATGACTTCAAGACTAACTCCTCTAAATCAATTACCAGTGGCTGCTGCCAGTGGTGCTTTTGCATGTCTTTCCGGGTTGGACTCAAGACGATAGTTACCGGATAAGGCGCAGCGGTCGGACTGAACGGGGGGTTCGTGCATACAGTCCAGCTTGGAGCGAACTGCCTACCCGGAACTGAGTGTCAGGCGTGGAATGAGACAAACGCGGCCATAACAGCGGAATGACACCGGTAAACCGAAAGGCAGGAACAGGAGAGCGCACGAGGGAGCCGCCAGGGGGAAACGCCTGGTATCTTTATAGTCCTGTCGGGTTTCGCCACCACTGATTTGAGCGTCAGATTTCGTGATGCTTGTCAGGGGGGCGGAGCCTATGGAAAAACGGCTTTGCCGCGGCCCTCTCACTTCCCTGTTAAGTATCTTCCTGGCATCTTCCAGGAAATCTCCGCCCCGTTCGTAAGCCATTTCCGCTCGCCGCAGTCGAACGACCGAGCGTAGCGAGTCAGTGAGCGAGGAAGCGGAATATATCCTGTATCACATATTCTGCTGACGCACCGGTGCAGCCTTTTTTCTCCTGCCACATGAAGCACTTCACTGACACCCTCATCAGTGCCAACATAGTAAGCCAGTATACACTCCGCTAGCGCTGAGGTCTGCCTCGTGAAGAAGGTGTTGCTGACTCATACCAGGCCTGAATCGCCCCATCATCCAGCCAGAAAGTGAGGGAGCCACGGTTGATGAGAGCTTTGTTGTAGGTGGACCAGTTGGTGATTTTGAACTTTTGCTTTGCCACGGAACGGTCTGCGTTGTCGGGAAGATGCGTGATCTGATCCTTCAACTCAGCAAAAGTTCGATTTATTCAACAAAGCCACGTTGTGTCTCAAAATCTCTGATGTTACATTGCACAAGATAAAAATATATCATCATGAACAATAAAACTGTCTGCTTACATAAACAGTAATACAAGGGGTGTTATGAGCCATATTCAACGGGAAACGTCTTGCTCGAGGCCGCGATTAAATTCCAACATGGATGCTGATTTATATGGGTATAAATGGGCTCGCGATAATGTCGGGCAATCAGGTGCGACAATCTATCGATTGTATGGGAAGCCCGATGCGCCAGAGTTGTTTCTGAAACATGGCAAAGGTAGCGTTGCCAATGATGTTACAGATGAGATGGTCAGACTAAACTGGCTGACGGAATTTATGCCTCTTCCGACCATCAAGCATTTTATCCGTACTCCTGATGATGCATGGTTACTCACCACTGCGATCCCCGGGAAAACAGCATTCCAGGTATTAGAAGAATATCCTGATTCAGGTGAAAATATTGTTGATGCGCTGGCAGTGTTCCTGCGCCGGTTGCATTCGATTCCTGTTTGTAATTGTCCTTTTAACAGCGATCGCGTATTTCGTCTCGCTCAGGCGCAATCACGAATGAATAACGGTTTGGTTGATGCGAGTGATTTTGATGACGAGCGTAATGGCTGGCCTGTTGAACAAGTCTGGAAAGAAATGCATAAGCTTTTGCCATTCTCACCGGATTCAGTCGTCACTCATGGTGATTTCTCACTTGATAACCTTATTTTTGACGAGGGGAAATTAATAGGTTGTATTGATGTTGGACGAGTCGGAATCGCAGACCGATACCAGGATCTTGCCATCCTATGGAACTGCCTCGGTGAGTTTTCTCCTTCATTACAGAAACGGCTTTTTCAAAAATATGGTATTGATAATCCTGATATGAATAAATTGCAGTTTCATTTGATGCTCGATGAGTTTTTCTAATCAGAATTGGTTAATTGGTTGTAACACTGGCAGAGCATTACGCTGACTTGACGGGACGGCGGCTTTGTTGAATAAATCGAACTTTTGCTGAGTTGAAGGATCAGATCACGCATCTTCCCGACAACGCAGACCGTTCCGTGGCAAAGCAAAAGTTCAAAATCACCAACTGGTCCACCTACAACAAAGCTCTCATCAACCGTGGCTCCCTCACTTTCTGGCTGGATGATGGGGCGATTCAGGCCTGGTATGAGTCAGCAACACCTTCTTCACGAGGCAGACCTCAGCGCTCAAAGATGCAGGGGTAAAAGCTAACCGCATCTTTACCGACAAGGCATCCGGCAGTTCAACAGATCGGGAAGGGCTGGATTTGCTGAGGATGAAGGTGGAGGAAGGTGATGTCATTCTGGTGAAGAAGCTCGACCGTCTTGGCCGCGACACCGCCGACATGATCCAACTGATAAAAGAGTTTGATGCTCAGGGTGTAGCGGTTCGGTTTATTGACGACGGGATCAGTACCGACGGTGATATGGGGCAAATGGTGGTCACCATCCTGTCGGCTGTGGCACAGGCTGAACGCCGGAGGATCCTAGAGCGCACGAATGAGGGCCGACAGGAAGCAAAGCTGAAAGGAATCAAATTTGGCCGCAGGCGTACCGTGGACAGGAACGTCGTGCTGACGCTTCATCAGAAGGGCACTGGTGCAACGGAAATTGCTCATCAGCTCAGTATTGCCCGCTCCACGGTTTATAAAATTCTTGAAGACGAAAGGGCCTCGTGATACGCCTATTTTTATAGGTTAATGTCATGATAATAATGGTTTCTTAGACGTCAGGTGGCACTTTTCGGGGAAATGTGCGCGGAACCCCTATTTGTTTATTTTTCTAAATACATTCAAATATGTATCCGCTCATGAGACAATAACCCTGATAAATGCTTCAATAATATTGAAAAAGGAAGAGTATGAGTATTCAACATTTCCGTGTCGCCCTTATTCCCTTTTTTGCGGCATTTTGCCTTCCTGTTTTTGCTCACCCAGAAACGCTGGTGAAAGTAAAAGATGCTGAAGATCAGTTGGGTGCACGAGTGGGTTACATCGAACTGGATCTCAACAGCGGTAAGATCCTTGAGAGTTTTCGCCCCGAAGAACGTTTTCCAATGATGAGCACTTTTAAAGTTCTGCTATGTGGCGCGGTATTATCCCGT |
| pACYC-araBAD-*tdc(C)* | GTTGACGATGCATAATGTGCCTGTCAAATGGACGAAGCAGGGATTCTGCAAACCCTATGCTACTCCGTCAAGCCGTCAATTGTCTGATTCGTTACCAATTATGACAACTTGACGGCTACATCATTCACTTTTTCTTCACAACCGGCACGGAACTCGCTCGGGCTGGCCCCGGTGCATTTTTTAAATACCCGCGAGAAATAGAGTTGATCGTCAAAACCAACATTGCGACCGACGGTGGCGATAGGCATCCGGGTGGTGCTCAAAAGCAGCTTCGCCTGGCTGATACGTTGGTCCTCGCGCCAGCTTAAGACGCTAATCCCTAACTGCTGGCGGAAAAGATGTGACAGACGCGACGGCGACAAGCAAACATGCTGTGCGACGCTGGCGATATCAAAATTGCTGTCTGCCAGGTGATCGCTGATGTACTGACAAGCCTCGCGTACCCGATTATCCATCGGTGGATGGAGCGACTCGTTAATCGCTTCCATGCGCCGCAGTAACAATTGCTCAAGCAGATTTATCGCCAGCAGCTCCGAATAGCGCCCTTCCCCTTGCCCGGCGTTAATGATTTGCCCAAACAGGTCGCTGAAATGCGGCTGGTGCGCTTCATCCGGGCGAAAGAACCCCGTATTGGCAAATATTGACGGCCAGTTAAGCCATTCATGCCAGTAGGCGCGCGGACGAAAGTAAACCCACTGGTGATACCATTCGCGAGCCTCCGGATGACGACCGTAGTGATGAATCTCTCCTGGCGGGAACAGCAAAATATCACCCGGTCGGCAAACAAATTCTCGTCCCTGATTTTTCACCACCCCCTGACCGCGAATGGTGAGATTGAGAATATAACCTTTCATTCCCAGCGGTCGGTCGATAAAAAAATCGAGATAACCGTTGGCCTCAATCGGCGTTAAACCCGCCACCAGATGGGCATTAAACGAGTATCCCGGCAGCAGGGGATCATTTTGCGCTTCAGCCATACTTTTCATACTCCCGCCATTCAGAGAAGAAACCAATTGTCCATATTGCATCAGACATTGCCGTCACTGCGTCTTTTACTGGCTCTTCTCGCTAACCAAACCGGTAACCCCGCTTATTAAAAGCATTCTGTAACAAAGCGGGACCAAAGCCATGACAAAAACGCGTAACAAAAGTGTCTATAATCACGGCAGAAAAGTCCACATTGATTATTTGCACGGCGTCACACTTTGCTATGCCATAGCATTTTTATCCATAAGATTAGCGGATCCTACCTGACGCTTTTTATCGCAACTCTCTACTGTTTCTCCATACCCGTTTTTTTGGGCTAGCAGGAGGAATTCACCATGGGCAGCATTGATAGCACCAACGTGGCGATGAGCAACAGCCCGGTGGGCGAATTTAAACCGCTGGAAGCGGAAGAATTTCGCAAACAAGCGCATCGCATGGTGGATTTTATTGCGGATTATTATAAAAACGTGGAAACCTATCCGGTGCTGAGCGAAGTGGAACCGGGCTATCTGCGCAAACGCATTCCGGAAACCGCGCCGTATCTGCCGGAACCGCTGGATGATATTATGAAAGATATTCAGAAAGATATTATTCCGGGCATGACCAACTGGATGAGCCCGAACTTTTATGCGTTTTTTCCGGCGACCGTGAGCAGCGCGGCGTTTCTGGGCGAAATGCTGAGCACCGCGCTGAACAGCGTGGGCTTTACCTGGGTGAGCAGCCCGGCGGCGACCGAACTGGAAATGATTGTGATGGATTGGCTGGCGCAGATTCTGAAACTGCCGAAGAGCTTTATGTTTAGCGGCACCGGCGGTGGCGTGATTCAGAACACCACGAGCGAAAGCATTCTGTGCACCATTATTGCGGCGCGCGAACGCGCGCTGGAAAAACTGGGCCCGGATAGCATTGGCAAACTGGTGTGCTATGGCAGCGATCAGACCCATACGATGTTTCCGAAAACCTGCAAACTGGCGGGCATTTATCCGAACAACATTCGCCTGATTCCGACCACCGTGGAAACCGATTTTGGCATTAGCCCGCAAGTGCTGCGCAAAATGGTGGAAGATGATGTGGCGGCGGGCTATGTGCCGCTGTTTCTGTGCGCGACCCTGGGCACCACGAGCACCACCGCGACCGATCCGGTTGATAGTCTGAGTGAAATTGCGAACGAATTTGGCATTTGGATTCATGTGGATGCGGCGTATGCGGGCAGCGCGTGCATTTGCCCGGAATTTCGCCATTATCTGGATGGCATTGAACGCGTGGATAGCCTGAGCCTGAGTCCGCATAAATGGCTGCTGGCGTATCTGGATTGCACCTGCCTGTGGGTGAAACAGCCGCATCTGTTACTGCGCGCGCTGACCACCAACCCGGAATATCTGAAAAACAAACAGAGCGATCTGGATAAAGTTGTGGATTTTAAAAATTGGCAGATTGCGACCGGCCGCAAATTTCGCAGCCTGAAACTGTGGCTGATTCTGCGCAGCTATGGCGTGGTGAACCTGCAGAGCCATATTCGCAGCGATGTGGCGATGGGCAAAATGTTTGAAGAATGGGTGCGCAGCGATAGCCGCTTTGAAATTGTGGTGCCGCGCAACTTTAGCCTGGTGTGCTTTCGCCTGAAACCGGATGTGAGCAGCCTGCATGTGGAAGAAGTGAACAAAAAACTGCTGGATATGCTGAACAGCACCGGCCGCGTGTATATGACCCATACCATTGTGGGCGGCATTTATATGCTGCGCCTGGCGGTGGGCAGCAGCCTGACCGAAGAACATCATGTGCGCCGCGTGTGGGATCTGATTCAGAAACTGACCGATGATCTGCTGAAAGAAGCGCACCATCACCATCACCATtgaCCATGGCAATGGCAACAACGTTGCGCAAACTATTAACTGGCGAACTACTTACTCTAGCTTCCCGGCAACAATTAATAGACTGGATGGAGGCGGATAAAGTTGCAGGACCACTTCTGCGCTCGGCCCTTCCGGCTGGCTGGTTTATTGCTGATAAATCTGGAGCCGGTGAGCGTGGGTCTCGCGGTATCATTGCAGCACTGGGGCCAGATGGTAAGCCCTCCCGTATCGTAGTTATCTACACGACGGGGAGTCAGGCAACTATGGATGAACGAAATAGACAGATCGCTGAGATAGGTGCCTCACTGATTAAGCATTGGTAACTGTCAGACCAAGTTTACTCATATATACTTTAGATTGATTTAAAACTTCATTTTTAATTTAAAAGGATCTAGGTGAAGATCCTTTTTGATAATCTCATGACCAAAATCCCTTAACGTGAGTTTTCGTTCCACTGAGCGTCAGACCCCTTAATAAGATGATCTTCTTGAGATCGTTTTGGTCTGCGCGTAATCTCTTGCTCTGAAAACGAAAAAACCGCCTTGCAGGGCGGTTTTTCGAAGGTTCTCTGAGCTACCAACTCTTTGAACCGAGGTAACTGGCTTGGAGGAGCGCAGTCACCAAAACTTGTCCTTTCAGTTTAGCCTTAACCGGCGCATGACTTCAAGACTAACTCCTCTAAATCAATTACCAGTGGCTGCTGCCAGTGGTGCTTTTGCATGTCTTTCCGGGTTGGACTCAAGACGATAGTTACCGGATAAGGCGCAGCGGTCGGACTGAACGGGGGGTTCGTGCATACAGTCCAGCTTGGAGCGAACTGCCTACCCGGAACTGAGTGTCAGGCGTGGAATGAGACAAACGCGGCCATAACAGCGGAATGACACCGGTAAACCGAAAGGCAGGAACAGGAGAGCGCACGAGGGAGCCGCCAGGGGGAAACGCCTGGTATCTTTATAGTCCTGTCGGGTTTCGCCACCACTGATTTGAGCGTCAGATTTCGTGATGCTTGTCAGGGGGGCGGAGCCTATGGAAAAACGGCTTTGCCGCGGCCCTCTCACTTCCCTGTTAAGTATCTTCCTGGCATCTTCCAGGAAATCTCCGCCCCGTTCGTAAGCCATTTCCGCTCGCCGCAGTCGAACGACCGAGCGTAGCGAGTCAGTGAGCGAGGAAGCGGAATATATCCTGTATCACATATTCTGCTGACGCACCGGTGCAGCCTTTTTTCTCCTGCCACATGAAGCACTTCACTGACACCCTCATCAGTGCCAACATAGTAAGCCAGTATACACTCCGCTAGCGCTGAGGTCTGCCTCGTGAAGAAGGTGTTGCTGACTCATACCAGGCCTGAATCGCCCCATCATCCAGCCAGAAAGTGAGGGAGCCACGGTTGATGAGAGCTTTGTTGTAGGTGGACCAGTTGGTGATTTTGAACTTTTGCTTTGCCACGGAACGGTCTGCGTTGTCGGGAAGATGCGTGATCTGATCCTTCAACTCAGCAAAAGTTCGATTTATTCAACAAAGCCACGTTGTGTCTCAAAATCTCTGATGTTACATTGCACAAGATAAAAATATATCATCATGAACAATAAAACTGTCTGCTTACATAAACAGTAATACAAGGGGTGTTATGAGCCATATTCAACGGGAAACGTCTTGCTCGAGGCCGCGATTAAATTCCAACATGGATGCTGATTTATATGGGTATAAATGGGCTCGCGATAATGTCGGGCAATCAGGTGCGACAATCTATCGATTGTATGGGAAGCCCGATGCGCCAGAGTTGTTTCTGAAACATGGCAAAGGTAGCGTTGCCAATGATGTTACAGATGAGATGGTCAGACTAAACTGGCTGACGGAATTTATGCCTCTTCCGACCATCAAGCATTTTATCCGTACTCCTGATGATGCATGGTTACTCACCACTGCGATCCCCGGGAAAACAGCATTCCAGGTATTAGAAGAATATCCTGATTCAGGTGAAAATATTGTTGATGCGCTGGCAGTGTTCCTGCGCCGGTTGCATTCGATTCCTGTTTGTAATTGTCCTTTTAACAGCGATCGCGTATTTCGTCTCGCTCAGGCGCAATCACGAATGAATAACGGTTTGGTTGATGCGAGTGATTTTGATGACGAGCGTAATGGCTGGCCTGTTGAACAAGTCTGGAAAGAAATGCATAAGCTTTTGCCATTCTCACCGGATTCAGTCGTCACTCATGGTGATTTCTCACTTGATAACCTTATTTTTGACGAGGGGAAATTAATAGGTTGTATTGATGTTGGACGAGTCGGAATCGCAGACCGATACCAGGATCTTGCCATCCTATGGAACTGCCTCGGTGAGTTTTCTCCTTCATTACAGAAACGGCTTTTTCAAAAATATGGTATTGATAATCCTGATATGAATAAATTGCAGTTTCATTTGATGCTCGATGAGTTTTTCTAATCAGAATTGGTTAATTGGTTGTAACACTGGCAGAGCATTACGCTGACTTGACGGGACGGCGGCTTTGTTGAATAAATCGAACTTTTGCTGAGTTGAAGGATCAGATCACGCATCTTCCCGACAACGCAGACCGTTCCGTGGCAAAGCAAAAGTTCAAAATCACCAACTGGTCCACCTACAACAAAGCTCTCATCAACCGTGGCTCCCTCACTTTCTGGCTGGATGATGGGGCGATTCAGGCCTGGTATGAGTCAGCAACACCTTCTTCACGAGGCAGACCTCAGCGCTCAAAGATGCAGGGGTAAAAGCTAACCGCATCTTTACCGACAAGGCATCCGGCAGTTCAACAGATCGGGAAGGGCTGGATTTGCTGAGGATGAAGGTGGAGGAAGGTGATGTCATTCTGGTGAAGAAGCTCGACCGTCTTGGCCGCGACACCGCCGACATGATCCAACTGATAAAAGAGTTTGATGCTCAGGGTGTAGCGGTTCGGTTTATTGACGACGGGATCAGTACCGACGGTGATATGGGGCAAATGGTGGTCACCATCCTGTCGGCTGTGGCACAGGCTGAACGCCGGAGGATCCTAGAGCGCACGAATGAGGGCCGACAGGAAGCAAAGCTGAAAGGAATCAAATTTGGCCGCAGGCGTACCGTGGACAGGAACGTCGTGCTGACGCTTCATCAGAAGGGCACTGGTGCAACGGAAATTGCTCATCAGCTCAGTATTGCCCGCTCCACGGTTTATAAAATTCTTGAAGACGAAAGGGCCTCGTGATACGCCTATTTTTATAGGTTAATGTCATGATAATAATGGTTTCTTAGACGTCAGGTGGCACTTTTCGGGGAAATGTGCGCGGAACCCCTATTTGTTTATTTTTCTAAATACATTCAAATATGTATCCGCTCATGAGACAATAACCCTGATAAATGCTTCAATAATATTGAAAAAGGAAGAGTATGAGTATTCAACATTTCCGTGTCGCCCTTATTCCCTTTTTTGCGGCATTTTGCCTTCCTGTTTTTGCTCACCCAGAAACGCTGGTGAAAGTAAAAGATGCTGAAGATCAGTTGGGTGCACGAGTGGGTTACATCGAACTGGATCTCAACAGCGGTAAGATCCTTGAGAGTTTTCGCCCCGAAGAACGTTTTCCAATGATGAGCACTTTTAAAGTTCTGCTATGTGGCGCGGTATTATCCCGT |
| pACYC-araBAD-*tdc(R)* | GTTGACGATGCATAATGTGCCTGTCAAATGGACGAAGCAGGGATTCTGCAAACCCTATGCTACTCCGTCAAGCCGTCAATTGTCTGATTCGTTACCAATTATGACAACTTGACGGCTACATCATTCACTTTTTCTTCACAACCGGCACGGAACTCGCTCGGGCTGGCCCCGGTGCATTTTTTAAATACCCGCGAGAAATAGAGTTGATCGTCAAAACCAACATTGCGACCGACGGTGGCGATAGGCATCCGGGTGGTGCTCAAAAGCAGCTTCGCCTGGCTGATACGTTGGTCCTCGCGCCAGCTTAAGACGCTAATCCCTAACTGCTGGCGGAAAAGATGTGACAGACGCGACGGCGACAAGCAAACATGCTGTGCGACGCTGGCGATATCAAAATTGCTGTCTGCCAGGTGATCGCTGATGTACTGACAAGCCTCGCGTACCCGATTATCCATCGGTGGATGGAGCGACTCGTTAATCGCTTCCATGCGCCGCAGTAACAATTGCTCAAGCAGATTTATCGCCAGCAGCTCCGAATAGCGCCCTTCCCCTTGCCCGGCGTTAATGATTTGCCCAAACAGGTCGCTGAAATGCGGCTGGTGCGCTTCATCCGGGCGAAAGAACCCCGTATTGGCAAATATTGACGGCCAGTTAAGCCATTCATGCCAGTAGGCGCGCGGACGAAAGTAAACCCACTGGTGATACCATTCGCGAGCCTCCGGATGACGACCGTAGTGATGAATCTCTCCTGGCGGGAACAGCAAAATATCACCCGGTCGGCAAACAAATTCTCGTCCCTGATTTTTCACCACCCCCTGACCGCGAATGGTGAGATTGAGAATATAACCTTTCATTCCCAGCGGTCGGTCGATAAAAAAATCGAGATAACCGTTGGCCTCAATCGGCGTTAAACCCGCCACCAGATGGGCATTAAACGAGTATCCCGGCAGCAGGGGATCATTTTGCGCTTCAGCCATACTTTTCATACTCCCGCCATTCAGAGAAGAAACCAATTGTCCATATTGCATCAGACATTGCCGTCACTGCGTCTTTTACTGGCTCTTCTCGCTAACCAAACCGGTAACCCCGCTTATTAAAAGCATTCTGTAACAAAGCGGGACCAAAGCCATGACAAAAACGCGTAACAAAAGTGTCTATAATCACGGCAGAAAAGTCCACATTGATTATTTGCACGGCGTCACACTTTGCTATGCCATAGCATTTTTATCCATAAGATTAGCGGATCCTACCTGACGCTTTTTATCGCAACTCTCTACTGTTTCTCCATACCCGTTTTTTTGGGCTAGCAGGAGGAATTCACCATGGGCAGCCTGGATACCAACCCGACCGCGTTTAGCGCGTTTCCGGCGGGCGAAGGCGAAACCTTTCAGCCGCTGAACGCGGATGATGTGCGCAGCTATCTGCATAAAGCGGTGGATTTTATTAGCGATTATTATAAAAGCGTGGAAAGCATGCCGGTGCTGCCGAACGTGAAACCGGGCTATCTGCAAGATGAACTGCGCGCGAGCCCGCCGACCTATAGCGCGCCGTTTGATGTGACCATGAAAGAACTGCGCAGCAGCGTGGTGCCGGGCATGACCCATTGGGCGAGCCCGAACTTTTTTGCGTTTTTTCCGAGCACCAACAGCGCGGCCGCGATTGCGGGCGATCTGATTGCGAGCGCGATGAACACCGTGGGCTTTACCTGGCAAGCGAGCCCGGCGGCGACCGAAATGGAAGTGCTGGCGCTGGATTGGCTGGCGCAGATGCTGAACCTGCCGACGAGCTTTATGAACCGCACCGGCGAAGGCCGCGGCACCGGCGGTGGCGTGATTCTGGGCACCACGAGCGAAGCGATGTTAGTGACGCTGGTTGCGGCGCGTGACGCCGCGCTGCGCCGCAGTGGTAGTGATGGCGTGGCGGGTCTGCATCGCCTGGCGGTTTATGCCGCGGATCAGACCCATAGCACCTTTTTTAAAGCGTGCCGCCTGGCGGGCTTTGATCCGGCGAACATTCGCAGCATTCCGACCGGCGCGGAAACCGATTATGGCTTAGATCCGGCGCGCCTGCTGGAAGCGATGCAAGCGGATGCGGATGCGGGCCTGGTGCCGACCTATGTGTGCGCGACCGTGGGTACCACGAGCAGTAACGCGGTTGATCCGGTGGGCGCGGTTGCGGATGTTGCGGCCCGCTTTGCGGCGTGGGTGCATGTGGATGCGGCGTATGCGGGCAGCGCGTGCATTTGCCCGGAATTTCGCCATCATCTGGATGGCGTGGAACGCGTGGATAGCATTAGCATGAGCCCGCATAAATGGCTGATGACCTGCCTGGATTGCACCTGCCTGTATGTGCGCGATACCCATCGCCTGACCGGCAGCCTGGAAACCAACCCGGAATATCTGAAAAACCATGCGAGCGATAGCGGCGAAGTGACCGATCTGAAAGATATGCAAGTGGGCGTGGGCCGCCGCTTTCGCGGCCTGAAACTGTGGATGGTGATGCGCACCTATGGCGTGGCGAAACTGCAAGAACATATTCGCAGCGATGTGGCGATGGCGAAAGTGTTTGAAGATCTGGTGCGCGGCGATGATCGCTTTGAAGTGGTTGTGCCGCGCAACTTTGCGCTGGTGTGCTTTCGCATTCGCGCGGGCGCGGGCGCGGCGGCCGCGACCGAAGAAGATGCGGATGAAGCGAACCGCGAACTGATGGAACGCCTGAACAAAACCGGCAAAGCGTATGTGGCGCATACCGTGGTGGGCGGCCGCTTTGTGCTGCGCTTTGCGGTGGGCAGCAGCCTGCAAGAAGAACATCATGTGCGCAGCGCGTGGGAACTGATTAAAAAAACCACGACCGAAATGATGAACTAACCATGGCAATGGCAACAACGTTGCGCAAACTATTAACTGGCGAACTACTTACTCTAGCTTCCCGGCAACAATTAATAGACTGGATGGAGGCGGATAAAGTTGCAGGACCACTTCTGCGCTCGGCCCTTCCGGCTGGCTGGTTTATTGCTGATAAATCTGGAGCCGGTGAGCGTGGGTCTCGCGGTATCATTGCAGCACTGGGGCCAGATGGTAAGCCCTCCCGTATCGTAGTTATCTACACGACGGGGAGTCAGGCAACTATGGATGAACGAAATAGACAGATCGCTGAGATAGGTGCCTCACTGATTAAGCATTGGTAACTGTCAGACCAAGTTTACTCATATATACTTTAGATTGATTTAAAACTTCATTTTTAATTTAAAAGGATCTAGGTGAAGATCCTTTTTGATAATCTCATGACCAAAATCCCTTAACGTGAGTTTTCGTTCCACTGAGCGTCAGACCCCTTAATAAGATGATCTTCTTGAGATCGTTTTGGTCTGCGCGTAATCTCTTGCTCTGAAAACGAAAAAACCGCCTTGCAGGGCGGTTTTTCGAAGGTTCTCTGAGCTACCAACTCTTTGAACCGAGGTAACTGGCTTGGAGGAGCGCAGTCACCAAAACTTGTCCTTTCAGTTTAGCCTTAACCGGCGCATGACTTCAAGACTAACTCCTCTAAATCAATTACCAGTGGCTGCTGCCAGTGGTGCTTTTGCATGTCTTTCCGGGTTGGACTCAAGACGATAGTTACCGGATAAGGCGCAGCGGTCGGACTGAACGGGGGGTTCGTGCATACAGTCCAGCTTGGAGCGAACTGCCTACCCGGAACTGAGTGTCAGGCGTGGAATGAGACAAACGCGGCCATAACAGCGGAATGACACCGGTAAACCGAAAGGCAGGAACAGGAGAGCGCACGAGGGAGCCGCCAGGGGGAAACGCCTGGTATCTTTATAGTCCTGTCGGGTTTCGCCACCACTGATTTGAGCGTCAGATTTCGTGATGCTTGTCAGGGGGGCGGAGCCTATGGAAAAACGGCTTTGCCGCGGCCCTCTCACTTCCCTGTTAAGTATCTTCCTGGCATCTTCCAGGAAATCTCCGCCCCGTTCGTAAGCCATTTCCGCTCGCCGCAGTCGAACGACCGAGCGTAGCGAGTCAGTGAGCGAGGAAGCGGAATATATCCTGTATCACATATTCTGCTGACGCACCG GTGCAGCCTTTTTTCTCCTGCCACATGAAGCACTTCACTGACACCCTCATCAGTGCCAACATAGTAAGCCAGTATACACTCCGCTAGCGCTGAGGTCTGCCTCGTGAAGAAGGTGTTGCTGACTCATACCAGGCCTGAATCGCCCCATCATCCAGCCAGAAAGTGAGGGAGCCACGGTTGATGAGAGCTTTGTTGTAGGTGGACCAGTTGGTGATTTTGAACTTTTGCTTTGCCACGGAACGGTCTGCGTTGTCGGGAAGATGCGTGATCTGATCCTTCAACTCAGCAAAAGTTCGATTTATTCAACAAAGCCACGTTGTGTCTCAAAATCTCTGATGTTACATTGCACAAGATAAAAATATATCATCATGAACAATAAAACTGTCTGCTTACATAAACAGTAATACAAGGGGTGTTATGAGCCATATTCAACGGGAAACGTCTTGCTCGAGGCCGCGATTAAATTCCAACATGGATGCTGATTTATATGGGTATAAATGGGCTCGCGATAATGTCGGGCAATCAGGTGCGACAATCTATCGATTGTATGGGAAGCCCGATGCGCCAGAGTTGTTTCTGAAACATGGCAAAGGTAGCGTTGCCAATGATGTTACAGATGAGATGGTCAGACTAAACTGGCTGACGGAATTTATGCCTCTTCCGACCATCAAGCATTTTATCCGTACTCCTGATGATGCATGGTTACTCACCACTGCGATCCCCGGGAAAACAGCATTCCAGGTATTAGAAGAATATCCTGATTCAGGTGAAAATATTGTTGATGCGCTGGCAGTGTTCCTGCGCCGGTTGCATTCGATTCCTGTTTGTAATTGTCCTTTTAACAGCGATCGCGTATTTCGTCTCGCTCAGGCGCAATCACGAATGAATAACGGTTTGGTTGATGCGAGTGATTTTGATGACGAGCGTAATGGCTGGCCTGTTGAACAAGTCTGGAAAGAAATGCATAAGCTTTTGCCATTCTCACCGGATTCAGTCGTCACTCATGGTGATTTCTCACTTGATAACCTTATTTTTGACGAGGGGAAATTAATAGGTTGTATTGATGTTGGACGAGTCGGAATCGCAGACCGATACCAGGATCTTGCCATCCTATGGAACTGCCTCGGTGAGTTTTCTCCTTCATTACAGAAACGGCTTTTTCAAAAATATGGTATTGATAATCCTGATATGAATAAATTGCAGTTTCATTTGATGCTCGATGAGTTTTTCTAATCAGAATTGGTTAATTGGTTGTAACACTGGCAGAGCATTACGCTGACTTGACGGGACGGCGGCTTTGTTGAATAAATCGAACTTTTGCTGAGTTGAAGGATCAGATCACGCATCTTCCCGACAACGCAGACCGTTCCGTGGCAAAGCAAAAGTTCAAAATCACCAACTGGTCCACCTACAACAAAGCTCTCATCAACCGTGGCTCCCTCACTTTCTGGCTGGATGATGGGGCGATTCAGGCCTGGTATGAGTCAGCAACACCTTCTTCACGAGGCAGACCTCAGCGCTCAAAGATGCAGGGGTAAAAGCTAACCGCATCTTTACCGACAAGGCATCCGGCAGTTCAACAGATCGGGAAGGGCTGGATTTGCTGAGGATGAAGGTGGAGGAAGGTGATGTCATTCTGGTGAAGAAGCTCGACCGTCTTGGCCGCGACACCGCCGACATGATCCAACTGATAAAAGAGTTTGATGCTCAGGGTGTAGCGGTTCGGTTTATTGACGACGGGATCAGTACCGACGGTGATATGGGGCAAATGGTGGTCACCATCCTGTCGGCTGTGGCACAGGCTGAACGCCGGAGGATCCTAGAGCGCACGAATGAGGGCCGACAGGAAGCAAAGCTGAAAGGAATCAAATTTGGCCGCAGGCGTACCGTGGACAGGAACGTCGTGCTGACGCTTCATCAGAAGGGCACTGGTGCAACGGAAATTGCTCATCAGCTCAGTATTGCCCGCTCCACGGTTTATAAAATTCTTGAAGACGAAAGGGCCTCGTGATACGCCTATTTTTATAGGTTAATGTCATGATAATAATGGTTTCTTAGACGTCAGGTGGCACTTTTCGGGGAAATGTGCGCGGAACCCCTATTTGTTTATTTTTCTAAATACATTCAAATATGTATCCGCTCATGAGACAATAACCCTGATAAATGCTTCAATAATATTGAAAAAGGAAGAGTATGAGTATTCAACATTTCCGTGTCGCCCTTATTCCCTTTTTTGCGGCATTTTGCCTTCCTGTTTTTGCTCACCCAGAAACGCTGGTGAAAGTAAAAGATGCTGAAGATCAGTTGGGTGCACGAGTGGGTTACATCGAACTGGATCTCAACAGCGGTAAGATCCTTGAGAGTTTTCGCCCCGAAGAACGTTTTCCAATGATGAGCACTTTTAAAGTTCTGCTATGTGGCGCGGTATTATCCCGT |
| pACYC-araBAD-*tdc(B)* | GTTGACGATGCATAATGTGCCTGTCAAATGGACGAAGCAGGGATTCTGCAAACCCTATGCTACTCCGTCAAGCCGTCAATTGTCTGATTCGTTACCAATTATGACAACTTGACGGCTACATCATTCACTTTTTCTTCACAACCGGCACGGAACTCGCTCGGGCTGGCCCCGGTGCATTTTTTAAATACCCGCGAGAAATAGAGTTGATCGTCAAAACCAACATTGCGACCGACGGTGGCGATAGGCATCCGGGTGGTGCTCAAAAGCAGCTTCGCCTGGCTGATACGTTGGTCCTCGCGCCAGCTTAAGACGCTAATCCCTAACTGCTGGCGGAAAAGATGTGACAGACGCGACGGCGACAAGCAAACATGCTGTGCGACGCTGGCGATATCAAAATTGCTGTCTGCCAGGTGATCGCTGATGTACTGACAAGCCTCGCGTACCCGATTATCCATCGGTGGATGGAGCGACTCGTTAATCGCTTCCATGCGCCGCAGTAACAATTGCTCAAGCAGATTTATCGCCAGCAGCTCCGAATAGCGCCCTTCCCCTTGCCCGGCGTTAATGATTTGCCCAAACAGGTCGCTGAAATGCGGCTGGTGCGCTTCATCCGGGCGAAAGAACCCCGTATTGGCAAATATTGACGGCCAGTTAAGCCATTCATGCCAGTAGGCGCGCGGACGAAAGTAAACCCACTGGTGATACCATTCGCGAGCCTCCGGATGACGACCGTAGTGATGAATCTCTCCTGGCGGGAACAGCAAAATATCACCCGGTCGGCAAACAAATTCTCGTCCCTGATTTTTCACCACCCCCTGACCGCGAATGGTGAGATTGAGAATATAACCTTTCATTCCCAGCGGTCGGTCGATAAAAAAATCGAGATAACCGTTGGCCTCAATCGGCGTTAAACCCGCCACCAGATGGGCATTAAACGAGTATCCCGGCAGCAGGGGATCATTTTGCGCTTCAGCCATACTTTTCATACTCCCGCCATTCAGAGAAGAAACCAATTGTCCATATTGCATCAGACATTGCCGTCACTGCGTCTTTTACTGGCTCTTCTCGCTAACCAAACCGGTAACCCCGCTTATTAAAAGCATTCTGTAACAAAGCGGGACCAAAGCCATGACAAAAACGCGTAACAAAAGTGTCTATAATCACGGCAGAAAAGTCCACATTGATTATTTGCACGGCGTCACACTTTGCTATGCCATAGCATTTTTATCCATAAGATTAGCGGATCCTACCTGACGCTTTTTATCGCAACTCTCTACTGTTTCTCCATACCCGTTTTTTTGGGCTAGCAGGAGGAATTCACCATGGGCATGAGCGAAAACCTGCAGCTGAGCGCGGAAGAAATGCGTCAGCTGGGCTATCAAGCGGTGGATCTGATTATTGATCATATGAACCATCTGAAAAGCAAACCGGTGAGCGAAACCATTGATAGCGATATTCTGCGCAACAAACTGACCGAAAGCATTCCGGAAAACGGCAGCGATCCGAAAGAACTGCTGCATTTTCTGAACCGCAACGTGTTTAATCAGATTACCCATGTGGATCATCCGCATTTTCTGGCGTTTGTGCCGGGCCCGAACAACTATGTGGGCGTGGTGGCGGATTTTCTGGCGAGCGGCTTTAACGTGTTTCCGACCGCGTGGATTGCGGGCGCGGGCGCGGAACAGATTGAACTGACCACCATTAACTGGCTGAAAAGCATGCTGGGCTTTCCGGATAGCGCGGAAGGCCTGTTTGTGAGCGGCGGCAGCATGGCGAACCTGACCGCGCTGACCGTGGCGCGCCAAGCGAAACTGAACAACGATATTGAAAACGCGGTGGTGTATTTTAGCGATCAGACCCATTTTAGCGTGGATCGCGCGCTGAAGGTGCTGGGCTTTAAACATCATCAGATTTGCCGCATTGAAACCGATGAACATCTGCGCATTAGCGTGAGCGCGCTGAAAAAACAGATTAAAGAAGATCGCACCAAAGGCAAAAAACCGTTTTGCGTGATTGCGAACGCGGGCACCACCAACTGCGGCGCGGTGGATAGCCTGAACGAACTGGCGGATCTGTGCAACGATGAAGATGTGTGGCTGCATGCGGATGGCAGCTATGGCGCGCCGGCGATTCTGAGCGAAAAAGGCAGCGCGATGCTGCAAGGCATTCATCGCGCGGATAGCCTGACCTTAGATCCGCATAAATGGCTGTTTCAGCCGTATGATGTGGGCTGCGTGCTGATTCGCAACAGTCAGTATCTGAGCAAAACCTTTCGCATGATGCCGGAATATATTAAAGATAGCGAAACCAACGTGGAAGGCGAAATTAACTTTGGCGAATGCGGCATTGAACTGAGCCGCCGCTTTCGCGCCCTGAAAGTTTGGCTGAGCTTTAAAGTGTTTGGCGTGGCGGCGTTTCGCCAAGCGATTGATCATGGCATTATGCTGGCGGAACAAGTGGAAGCGTTTCTGGGCAAAGCGAAAGATTGGGAAGTGGTGACCCCGGCGCAGCTGGGCATTGTGACCTTTCGCTATATTCCGAGCGAACTGGCGAGCACCGATACCATTAACGAAATTAACAAAAAACTGGTGAAAGAAATTACCCATCGCGGCTTTGCGATGCTGAGCACCACCGAACTGAAAGAAAAAGTGGTGATTCGCCTGTGCAGCATTAACCCGCGCACCACGACCGAAGAAATGCTGCAGATTATGATGAAAATTAAAGCGCTGGCGGAAGAAGTGAGCATTAGCTATCCGTGCGTGGCGGAATAACCATGGCAATGGCAACAACGTTGCGCAAACTATTAACTGGCGAACTACTTACTCTAGCTTCCCGGCAACAATTAATAGACTGGATGGAGGCGGATAAAGTTGCAGGACCACTTCTGCGCTCGGCCCTTCCGGCTGGCTGGTTTATTGCTGATAAATCTGGAGCCGGTGAGCGTGGGTCTCGCGGTATCATTGCAGCACTGGGGCCAGATGGTAAGCCCTCCCGTATCGTAGTTATCTACACGACGGGGAGTCAGGCAACTATGGATGAACGAAATAGACAGATCGCTGAGATAGGTGCCTCACTGATTAAGCATTGGTAACTGTCAGACCAAGTTTACTCATATATACTTTAGATTGATTTAAAACTTCATTTTTAATTTAAAAGGATCTAGGTGAAGATCCTTTTTGATAATCTCATGACCAAAATCCCTTAACGTGAGTTTTCGTTCCACTGAGCGTCAGACCCCTTAATAAGATGATCTTCTTGAGATCGTTTTGGTCTGCGCGTAATCTCTTGCTCTGAAAACGAAAAAACCGCCTTGCAGGGCGGTTTTTCGAAGGTTCTCTGAGCTACCAACTCTTTGAACCGAGGTAACTGGCTTGGAGGAGCGCAGTCACCAAAACTTGTCCTTTCAGTTTAGCCTTAACCGGCGCATGACTTCAAGACTAACTCCTCTAAATCAATTACCAGTGGCTGCTGCCAGTGGTGCTTTTGCATGTCTTTCCGGGTTGGACTCAAGACGATAGTTACCGGATAAGGCGCAGCGGTCGGACTGAACGGGGGGTTCGTGCATACAGTCCAGCTTGGAGCGAACTGCCTACCCGGAACTGAGTGTCAGGCGTGGAATGAGACAAACGCGGCCATAACAGCGGAATGACACCGGTAAACCGAAAGGCAGGAACAGGAGAGCGCACGAGGGAGCCGCCAGGGGGAAACGCCTGGTATCTTTATAGTCCTGTCGGGTTTCGCCACCACTGATTTGAGCGTCAGATTTCGTGATGCTTGTCAGGGGGGCGGAGCCTATGGAAAAACGGCTTTGCCGCGGCCCTCTCACTTCCCTGTTAAGTATCTTCCTGGCATCTTCCAGGAAATCTCCGCCCCGTTCGTAAGCCATTTCCGCTCGCCGCAGTCGAACGACCGAGCGTAGCGAGTCAGTGAGCGAGGAAGCGGAATATATCCTGTATCACATATTCTGCTGACGCACCGGTGCAGCCTTTTTTCTCCTGCCACATGAAGCACTTCACTGACACCCTCATCAGTGCCAACATAGTAAGCCAGTATACACTCCGCTAGCGCTGAGGTCTGCCTCGTGAAGAAGGTGTTGCTGACTCATACCAGGCCTGAATCGCCCCATCATCCAGCCAGAAAGTGAGGGAGCCACGGTTGATGAGAGCTTTGTTGTAGGTGGACCAGTTGGTGATTTTGAACTTTTGCTTTGCCACGGAACGGTCTGCGTTGTCGGGAAGATGCGTGATCTGATCCTTCAACTCAGCAAAAGTTCGATTTATTCAACAAAGCCACGTTGTGTCTCAAAATCTCTGATGTTACATTGCACAAGATAAAAATATATCATCATGAACAATAAAACTGTCTGCTTACATAAACAGTAATACAAGGGGTGTTATGAGCCATATTCAACGGGAAACGTCTTGCTCGAGGCCGCGATTAAATTCCAACATGGATGCTGATTTATATGGGTATAAATGGGCTCGCGATAATGTCGGGCAATCAGGTGCGACAATCTATCGATTGTATGGGAAGCCCGATGCGCCAGAGTTGTTTCTGAAACATGGCAAAGGTAGCGTTGCCAATGATGTTACAGATGAGATGGTCAGACTAAACTGGCTGACGGAATTTATGCCTCTTCCGACCATCAAGCATTTTATCCGTACTCCTGATGATGCATGGTTACTCACCACTGCGATCCCCGGGAAAACAGCATTCCAGGTATTAGAAGAATATCCTGATTCAGGTGAAAATATTGTTGATGCGCTGGCAGTGTTCCTGCGCCGGTTGCATTCGATTCCTGTTTGTAATTGTCCTTTTAACAGCGATCGCGTATTTCGTCTCGCTCAGGCGCAATCACGAATGAATAACGGTTTGGTTGATGCGAGTGATTTTGATGACGAGCGTAATGGCTGGCCTGTTGAACAAGTCTGGAAAGAAATGCATAAGCTTTTGCCATTCTCACCGGATTCAGTCGTCACTCATGGTGATTTCTCACTTGATAACCTTATTTTTGACGAGGGGAAATTAATAGGTTGTATTGATGTTGGACGAGTCGGAATCGCAGACCGATACCAGGATCTTGCCATCCTATGGAACTGCCTCGGTGAGTTTTCTCCTTCATTACAGAAACGGCTTTTTCAAAAATATGGTATTGATAATCCTGATATGAATAAATTGCAGTTTCATTTGATGCTCGATGAGTTTTTCTAATCAGAATTGGTTAATTGGTTGTAACACTGGCAGAGCATTACGCTGACTTGACGGGACGGCGGCTTTGTTGAATAAATCGAACTTTTGCTGAGTTGAAGGATCAGATCACGCATCTTCCCGACAACGCAGACCGTTCCGTGGCAAAGCAAAAGTTCAAAATCACCAACTGGTCCACCTACAACAAAGCTCTCATCAACCGTGGCTCCCTCACTTTCTGGCTGGATGATGGGGCGATTCAGGCCTGGTATGAGTCAGCAACACCTTCTTCACGAGGCAGACCTCAGCGCTCAAAGATGCAGGGGTAAAAGCTAACCGCATCTTTACCGACAAGGCATCCGGCAGTTCAACAGATCGGGAAGGGCTGGATTTGCTGAGGATGAAGGTGGAGGAAGGTGATGTCATTCTGGTGAAGAAGCTCGACCGTCTTGGCCGCGACACCGCCGACATGATCCAACTGATAAAAGAGTTTGATGCTCAGGGTGTAGCGGTTCGGTTTATTGACGACGGGATCAGTACCGACGGTGATATGGGGCAAATGGTGGTCACCATCCTGTCGGCTGTGGCACAGGCTGAACGCCGGAGGATCCTAGAGCGCACGAATGAGGGCCGACAGGAAGCAAAGCTGAAAGGAATCAAATTTGGCCGCAGGCGTACCGTGGACAGGAACGTCGTGCTGACGCTTCATCAGAAGGGCACTGGTGCAACGGAAATTGCTCATCAGCTCAGTATTGCCCGCTCCACGGTTTATAAAATTCTTGAAGACGAAAGGGCCTCGTGATACGCCTATTTTTATAGGTTAATGTCATGATAATAATGGTTTCTTAGACGTCAGGTGGCACTTTTCGGGGAAATGTGCGCGGAACCCCTATTTGTTTATTTTTCTAAATACATTCAAATATGTATCCGCTCATGAGACAATAACCCTGATAAATGCTTCAATAATATTGAAAAAGGAAGAGTATGAGTATTCAACATTTCCGTGTCGCCCTTATTCCCTTTTTTGCGGCATTTTGCCTTCCTGTTTTTGCTCACCCAGAAACGCTGGTGAAAGTAAAAGATGCTGAAGATCAGTTGGGTGCACGAGTGGGTTACATCGAACTGGATCTCAACAGCGGTAAGATCCTTGAGAGTTTTCGCCCCGAAGAACGTTTTCCAATGATGAGCACTTTTAAAGTTCTGCTATGTGGCGCGGTATTATCCCGT |
| pKD46 | CATCGATTTATTATGACAACTTGACGGCTACATCATTCACTTTTTCTTCACAACCGGCACGGAACTCGCTCGGGCTGGCCCCGGTGCATTTTTTAAATACCCGCGAGAAATAGAGTTGATCGTCAAAACCAACATTGCGACCGACGGTGGCGATAGGCATCCGGGTGGTGCTCAAAAGCAGCTTCGCCTGGCTGATACGTTGGTCCTCGCGCCAGCTTAAGACGCTAATCCCTAACTGCTGGCGGAAAAGATGTGACAGACGCGACGGCGACAAGCAAACATGCTGTGCGACGCTGGCGATATCAAAATTGCTGTCTGCCAGGTGATCGCTGATGTACTGACAAGCCTCGCGTACCCGATTATCCATCGGTGGATGGAGCGACTCGTTAATCGCTTCCATGCGCCGCAGTAACAATTGCTCAAGCAGATTTATCGCCAGCAGCTCCGAATAGCGCCCTTCCCCTTGCCCGGCGTTAATGATTTGCCCAAACAGGTCGCTGAAATGCGGCTGGTGCGCTTCATCCGGGCGAAAGAACCCCGTATTGGCAAATATTGACGGCCAGTTAAGCCATTCATGCCAGTAGGCGCGCGGACGAAAGTAAACCCACTGGTGATACCATTCGCGAGCCTCCGGATGACGACCGTAGTGATGAATCTCTCCTGGCGGGAACAGCAAAATATCACCCGGTCGGCAAACAAATTCTCGTCCCTGATTTTTCACCACCCCCTGACCGCGAATGGTGAGATTGAGAATATAACCTTTCATTCCCAGCGGTCGGTCGATAAAAAAATCGAGATAACCGTTGGCCTCAATCGGCGTTAAACCCGCCACCAGATGGGCATTAAACGAGTATCCCGGCAGCAGGGGATCATTTTGCGCTTCAGCCATACTTTTCATACTCCCGCCATTCAGAGAAGAAACCAATTGTCCATATTGCATCAGACATTGCCGTCACTGCGTCTTTTACTGGCTCTTCTCGCTAACCAAACCGGTAACCCCGCTTATTAAAAGCATTCTGTAACAAAGCGGGACCAAAGCCATGACAAAAACGCGTAACAAAAGTGTCTATAATCACGGCAGAAAAGTCCACATTGATTATTTGCACGGCGTCACACTTTGCTATGCCATAGCATTTTTATCCATAAGATTAGCGGATCCTACCTGACGCTTTTTATCGCAACTCTCTACTGTTTCTCCATACCCGTTTTTTTGGGAATTCGAGCTCTAAGGAGGTTATAAAAAATGGATATTAATACTGAAACTGAGATCAAGCAAAAGCATTCACTAACCCCCTTTCCTGTTTTCCTAATCAGCCCGGCATTTCGCGGGCGATATTTTCACAGCTATTTCAGGAGTTCAGCCATGAACGCTTATTACATTCAGGATCGTCTTGAGGCTCAGAGCTGGGCGCGTCACTACCAGCAGCTCGCCCGTGAAGAGAAAGAGGCAGAACTGGCAGACGACATGGAAAAAGGCCTGCCCCAGCACCTGTTTGAATCGCTATGCATCGATCATTTGCAACGCCACGGGGCCAGCAAAAAATCCATTACCCGTGCGTTTGATGACGATGTTGAGTTTCAGGAGCGCATGGCAGAACACATCCGGTACATGGTTGAAACCATTGCTCACCACCAGGTTGATATTGATTCAGAGGTATAAAACGAATGAGTACTGCACTCGCAACGCTGGCTGGGAAGCTGGCTGAACGTGTCGGCATGGATTCTGTCGACCCACAGGAACTGATCACCACTCTTCGCCAGACGGCATTTAAAGGTGATGCCAGCGATGCGCAGTTCATCGCATTACTGATCGTTGCCAACCAGTACGGCCTTAATCCGTGGACGAAAGAAATTTACGCCTTTCCTGATAAGCAGAATGGCATCGTTCCGGTGGTGGGCGTTGATGGCTGGTCCCGCATCATCAATGAAAACCAGCAGTTTGATGGCATGGACTTTGAGCAGGACAATGAATCCTGTACATGCCGGATTTACCGCAAGGACCGTAATCATCCGATCTGCGTTACCGAATGGATGGATGAATGCCGCCGCGAACCATTCAAAACTCGCGAAGGCAGAGAAATCACGGGGCCGTGGCAGTCGCATCCCAAACGGATGTTACGTCATAAAGCCATGATTCAGTGTGCCCGTCTGGCCTTCGGATTTGCTGGTATCTATGACAAGGATGAAGCCGAGCGCATTGTCGAAAATACTGCATACACTGCAGAACGTCAGCCGGAACGCGACATCACTCCGGTTAACGATGAAACCATGCAGGAGATTAACACTCTGCTGATCGCCCTGGATAAAACATGGGATGACGACTTATTGCCGCTCTGTTCCCAGATATTTCGCCGCGACATTCGTGCATCGTCAGAACTGACACAGGCCGAAGCAGTAAAAGCTCTTGGATTCCTGAAACAGAAAGCCGCAGAGCAGAAGGTGGCAGCATGACACCGGACATTATCCTGCAGCGTACCGGGATCGATGTGAGAGCTGTCGAACAGGGGGATGATGCGTGGCACAAATTACGGCTCGGCGTCATCACCGCTTCAGAAGTTCACAACGTGATAGCAAAACCCCGCTCCGGAAAGAAGTGGCCTGACATGAAAATGTCCTACTTCCACACCCTGCTTGCTGAGGTTTGCACCGGTGTGGCTCCGGAAGTTAACGCTAAAGCACTGGCCTGGGGAAAACAGTACGAGAACGACGCCAGAACCCTGTTTGAATTCACTTCCGGCGTGAATGTTACTGAATCCCCGATCATCTATCGCGACGAAAGTATGCGTACCGCCTGCTCTCCCGATGGTTTATGCAGTGACGGCAACGGCCTTGAACTGAAATGCCCGTTTACCTCCCGGGATTTCATGAAGTTCCGGCTCGGTGGTTTCGAGGCCATAAAGTCAGCTTACATGGCCCAGGTGCAGTACAGCATGTGGGTGACGCGAAAAAATGCCTGGTACTTTGCCAACTATGACCCGCGTATGAAGCGTGAAGGCCTGCATTATGTCGTGATTGAGCGGGATGAAAAGTACATGGCGAGTTTTGACGAGATCGTGCCGGAGTTCATCGAAAAAATGGACGAGGCACTGGCTGAAATTGGTTTTGTATTTGGGGAGCAATGGCGATGACGCATCCTCACGATAATATCCGGGTAGGCGCAATCACTTTCGTCTACTCCGTTACAAAGCGAGGCTGGGTATTTCCCGGCCTTTCTGTTATCCGAAATCCACTGAAAGCACAGCGGCTGGCTGAGGAGATAAATAATAAACGAGGGGCTGTATGCACAAAGCATCTTCTGTTGAGTTAAGAACGAGTATCGAGATGGCACATAGCCTTGCTCAAATTGGAATCAGGTTTGTGCCAATACCAGTAGAAACAGACGAAGAATCCATGGGTATGGACAGTTTTCCCTTTGATATGTAACGGTGAACAGTTGTTCTACTTTTGTTTGTTAGTCTTGATGCTTCACTGATAGATACAAGAGCCATAAGAACCTCAGATCCTTCCGTATTTAGCCAGTATGTTCTCTAGTGTGGTTCGTTGTTTTTGCGTGAGCCATGAGAACGAACCATTGAGATCATACTTACTTTGCATGTCACTCAAAAATTTTGCCTCAAAACTGGTGAGCTGAATTTTTGCAGTTAAAGCATCGTGTAGTGTTTTTCTTAGTCCGTTACGTAGGTAGGAATCTGATGTAATGGTTGTTGGTATTTTGTCACCATTCATTTTTATCTGGTTGTTCTCAAGTTCGGTTACGAGATCCATTTGTCTATCTAGTTCAACTTGGAAAATCAACGTATCAGTCGGGCGGCCTCGCTTATCAACCACCAATTTCATATTGCTGTAAGTGTTTAAATCTTTACTTATTGGTTTCAAAACCCATTGGTTAAGCCTTTTAAACTCATGGTAGTTATTTTCAAGCATTAACATGAACTTAAATTCATCAAGGCTAATCTCTATATTTGCCTTGTGAGTTTTCTTTTGTGTTAGTTCTTTTAATAACCACTCATAAATCCTCATAGAGTATTTGTTTTCAAAAGACTTAACATGTTCCAGATTATATTTTATGAATTTTTTTAACTGGAAAAGATAAGGCAATATCTCTTCACTAAAAACTAATTCTAATTTTTCGCTTGAGAACTTGGCATAGTTTGTCCACTGGAAAATCTCAAAGCCTTTAACCAAAGGATTCCTGATTTCCACAGTTCTCGTCATCAGCTCTCTGGTTGCTTTAGCTAATACACCATAAGCATTTTCCCTACTGATGTTCATCATCTGAGCGTATTGGTTATAAGTGAACGATACCGTCCGTTCTTTCCTTGTAGGGTTTTCAATCGTGGGGTTGAGTAGTGCCACACAGCATAAAATTAGCTTGGTTTCATGCTCCGTTAAGTCATAGCGACTAATCGCTAGTTCATTTGCTTTGAAAACAACTAATTCAGACATACATCTCAATTGGTCTAGGTGATTTTAATCACTATACCAATTGAGATGGGCTAGTCAATGATAATTACTAGTCCTTTTCCTTTGAGTTGTGGGTATCTGTAAATTCTGCTAGACCTTTGCTGGAAAACTTGTAAATTCTGCTAGACCCTCTGTAAATTCCGCTAGACCTTTGTGTGTTTTTTTTGTTTATATTCAAGTGGTTATAATTTATAGAATAAAGAAAGAATAAAAAAAGATAAAAAGAATAGATCCCAGCCCTGTGTATAACTCACTACTTTAGTCAGTTCCGCAGTATTACAAAAGGATGTCGCAAACGCTGTTTGCTCCTCTACAAAACAGACCTTAAAACCCTAAAGGCTTAAGTAGCACCCTCGCAAGCTCGGTTGCGGCCGCAATCGGGCAAATCGCTGAATATTCCTTTTGTCTCCGACCATCAGGCACCTGAGTCGCTGTCTTTTTCGTGACATTCAGTTCGCTGCGCTCACGGCTCTGGCAGTGAATGGGGGTAAATGGCACTACAGGCGCCTTTTATGGATTCATGCAAGGAAACTACCCATAATACAAGAAAAGCCCGTCACGGGCTTCTCAGGGCGTTTTATGGCGGGTCTGCTATGTGGTGCTATCTGACTTTTTGCTGTTCAGCAGTTCCTGCCCTCTGATTTTCCAGTCTGACCACTTCGGATTATCCCGTGACAGGTCATTCAGACTGGCTAATGCACCCAGTAAGGCAGCGGTATCATCAACGGGGTCTGACGCTCAGTGGAACGAAAACTCACGTTAAGGGATTTTGGTCATGAGATTATCAAAAAGGATCTTCACCTAGATCCTTTTAAATTAAAAATGAAGTTTTAAATCAATCTAAAGTATATATGAGTAAACTTGGTCTGACAGTTACCAATGCTTAATCAGTGAGGCACCTATCTCAGCGATCTGTCTATTTCGTTCATCCATAGTTGCCTGACTCCCCGTCGTGTAGATAACTACGATACGGGAGGGCTTACCATCTGGCCCCAGTGCTGCAATGATACCGCGAGACCCACGCTCACCGGCTCCAGATTTATCAGCAATAAACCAGCCAGCCGGAAGGGCCGAGCGCAGAAGTGGTCCTGCAACTTTATCCGCCTCCATCCAGTCTATTAATTGTTGCCGGGAAGCTAGAGTAAGTAGTTCGCCAGTTAATAGTTTGCGCAACGTTGTTGCCATTGCTACAGGCATCGTGGTGTCACGCTCGTCGTTTGGTATGGCTTCATTCAGCTCCGGTTCCCAACGATCAAGGCGAGTTACATGATCCCCCATGTTGTGCAAAAAAGCGGTTAGCTCCTTCGGTCCTCCGATCGTTGTCAGAAGTAAGTTGGCCGCAGTGTTATCACTCATGGTTATGGCAGCACTGCATAATTCTCTTACTGTCATGCCATCCGTAAGATGCTTTTCTGTGACTGGTGAGTACTCAACCAAGTCATTCTGAGAATAGTGTATGCGGCGACCGAGTTGCTCTTGCCCGGCGTCAATACGGGATAATACCGCGCCACATAGCAGAACTTTAAAAGTGCTCATCATTGGAAAACGTTCTTCGGGGCGAAAACTCTCAAGGATCTTACCGCTGTTGAGATCCAGTTCGATGTAACCCACTCGTGCACCCAACTGATCTTCAGCATCTTTTACTTTCACCAGCGTTTCTGGGTGAGCAAAAACAGGAAGGCAAAATGCCGCAAAAAAGGGAATAAGGGCGACACGGAAATGTTGAATACTCATACTCTTCCTTTTTCAATATTATTGAAGCATTTATCAGGGTTATTGTCTCATGAGCGGATACATATTTGAATGTATTTAGAAAAATAAACAAATAGGGGTTCCGCGCACATTTCCCCGAAAAGTGCCACCTG |
| pKD3 | AGATTGCAGCATTACACGTCTTGAGCGATTGTGTAGGCTGGAGCTGCTTCGAAGTTCCTATACTTTCTAGAGAATAGGAACTTCGGAATAGGAACTTCATTTAAATGGCGCGCCTTACGCCCCGCCCTGCCACTCATCGCAGTACTGTTGTATTCATTAAGCATCTGCCGACATGGAAGCCATCACAAACGGCATGATGAACCTGAATCGCCAGCGGCATCAGCACCTTGTCGCCTTGCGTATAATATTTGCCCATGGTGAAAACGGGGGCGAAGAAGTTGTCCATATTGGCCACGTTTAAATCAAAACTGGTGAAACTCACCCAGGGATTGGCTGAGACGAAAAACATATTCTCAATAAACCCTTTAGGGAAATAGGCCAGGTTTTCACCGTAACACGCCACATCTTGCGAATATATGTGTAGAAACTGCCGGAAATCGTCGTGGTATTCACTCCAGAGCGATGAAAACGTTTCAGTTTGCTCATGGAAAACGGTGTAACAAGGGTGAACACTATCCCATATCACCAGCTCACCGTCTTTCATTGCCATACGTAATTCCGGATGAGCATTCATCAGGCGGGCAAGAATGTGAATAAAGGCCGGATAAAACTTGTGCTTATTTTTCTTTACGGTCTTTAAAAAGGCCGTAATATCCAGCTGAACGGTCTGGTTATAGGTACATTGAGCAACTGACTGAAATGCCTCAAAATGTTCTTTACGATGCCATTGGGATATATCAACGGTGGTATATCCAGTGATTTTTTTCTCCATTTTAGCTTCCTTAGCTCCTGAAAATCTCGACAACTCAAAAAATACGCCCGGTAGTGATCTTATTTCATTATGGTGAAAGTTGGAACCTCTTACGTGCCGATCAACGTCTCATTTTCGCCAAAAGTTGGCCCAGGGCTTCCCGGTATCAACAGGGACACCAGGATTTATTTATTCTGCGAAGTGATCTTCCGTCACAGGTAGGCGCGCCGAAGTTCCTATACTTTCTAGAGAATAGGAACTTCGGAATAGGAACTAAGGAGGATATTCATATGGACCATGGCTAATTCCCATGTCAGCCGTTAAGTGTTCCTGTGTCACTGAAAATTGCTTTGAGAGGCTCTAAGGGCTTCTCAGTGCGTTACATCCCTGGCTTGTTGTCCACAACCGTTAAACCTTAAAAGCTTTAAAAGCCTTATATATTCTTTTTTTTCTTATAAAACTTAAAACCTTAGAGGCTATTTAAGTTGCTGATTTATATTAATTTTATTGTTCAAACATGAGAGCTTAGTACGTGAAACATGAGAGCTTAGTACGTTAGCCATGAGAGCTTAGTACGTTAGCCATGAGGGTTTAGTTCGTTAAACATGAGAGCTTAGTACGTTAAACATGAGAGCTTAGTACGTGAAACATGAGAGCTTAGTACGTACTATCAACAGGTTGAACTGCGGATCTTGCGGCCGCAAAAATTAAAAATGAAGTTTTAAATCAATCTAAAGTATATATGAGTAAACTTGGTCTGACAGTTACCAATGCTTAATCAGTGAGGCACCTATCTCAGCGATCTGTCTATTTCGTTCATCCATAGTTGCCTGACTCCCCGTCGTGTAGATAACTACGATACGGGAGGGCTTACCATCTGGCCCCAGTGCTGCAATGATACCGCGAGACCCACGCTCACCGGCTCCAGATTTATCAGCAATAAACCAGCCAGCCGGAAGGGCCGAGCGCAGAAGTGGTCCTGCAACTTTATCCGCCTCCATCCAGTCTATTAATTGTTGCCGGGAAGCTAGAGTAAGTAGTTCGCCAGTTAATAGTTTGCGCAACGTTGTTGCCATTGCTACAGGCATCGTGGTGTCACGCTCGTCGTTTGGTATGGCTTCATTCAGCTCCGGTTCCCAACGATCAAGGCGAGTTACATGATCCCCCATGTTGTGCAAAAAAGCGGTTAGCTCCTTCGGTCCTCCGATCGTTGTCAGAAGTAAGTTGGCCGCAGTGTTATCACTCATGGTTATGGCAGCACTGCATAATTCTCTTACTGTCATGCCATCCGTAAGATGCTTTTCTGTGACTGGTGAGTACTCAACCAAGTCATTCTGAGAATAGTGTATGCGGCGACCGAGTTGCTCTTGCCCGGCGTCAATACGGGATAATACCGCGCCACATAGCAGAACTTTAAAAGTGCTCATCATTGGAAAACGTTCTTCGGGGCGAAAACTCTCAAGGATCTTACCGCTGTTGAGATCCAGTTCGATGTAACCCACTCGTGCACCCAACTGATCTTCAGCATCTTTTACTTTCACCAGCGTTTCTGGGTGAGCAAAAACAGGAAGGCAAAATGCCGCAAAAAAGGGAATAAGGGCGACACGGAAATGTTGAATACTCATACTCTTCCTTTTTCAATATTATTGAAGCATTTATCAGGGTTATTGTCTCATGAGCGGATACATATTTGAATGTATTTAGAAAAATAAACAAATAGGGGTTCCGCGCACATTTCCCCGAAAAGTGCCACCTGCATCGATGGCCCCCCGATGGTAGTGTGGGGTCTCCCCATGCGAGAGTAGGGAACTGCCAGGCATCAAATAAAACGAAAGGCTCAGTCGAAAGACTGGGCCTTTCGTTTTATCTGTTGTTTGTCGGTGAACGCTCTCCTGAGTAGGACAAATCCGCCGGGAGCGGATTTGAACGTTGCGAAGCAACGGCCCGGAGGGTGGCGGGCAGGACGCCCGCCATAAACTGCCAGGCATCAAATTAAGCAGAAGGCCATCCTGACGGATGGCCTTTTTGCGTGGCCAGTGCCAAGCTTGCATGC |
| pCP20 | GAGACACAACGTGGCTTTGTTGAATAAATCGAACTTTTGCTGAGTTGAAGGATCAGATCACGCATCTTCCCGACAACGCAGACCGTTCCGTGGCAAAGCAAAAGTTCAAAATCACCAACTGGTCCACCTACAACAAAGCTCTCATCAACCGTGGCTCCCTCACTTTCTGGCTGGATGATGGGGCGATTCAGGCCTGGTATGAGTCAGCAACACCTTCTTCACGAGGCAGACCTCAGCGCCACAGGTGCGGTTGCTGGCGCTAACCGTTTTTATCAGGCTCTGGGAGGCAGAATAAATGATCATATCGTCAATTATTACCTCCACGGGGAGAGCCTGAGCAAACTGGCCTCAGGCATTTGAGAAGCACACGGTCACACTGCTTCCGGTAGTCAATAAACCGGTAAACCAGCAATAGACATAAGCGGCTATTTAACGACCCTGCCCTGAACCGACGACCGGGTCGAATTTGCTTTCGAATTTCTGCCATTCATCCGCTTATTATCACTTATTCAGGCGTAGCAACCAGGCGTTTAAGGGCACCAATAACTGCCTTAAAAAAATTACGCCCCGCCCTGCCACTCATCGCAGTACTGTTGTAATTCATTAAGCATTCTGCCGACATGGAAGCCATCACAAACGGCATGATGAACCTGAATCGCCAGCGGCATCAGCACCTTGTCGCCTTGCGTATAATATTTGCCCATGGTGAAAACGGGGGCGAAGAAGTTGTCCATATTGGCCACGTTTAAATCAAAACTGGTGAAACTCACCCAGGGATTGGCTGAGACGAAAAACATATTCTCAATAAACCCTTTAGGGAAATAGGCCAGGTTTTCACCGTAACACGCCACATCTTGCGAATATATGTGTAGAAACTGCCGGAAATCGTCGTGGTATTCACTCCAGAGCGATGAAAACGTTTCAGTTTGCTCATGGAAAACGGTGTAACAAGGGTGAACACTATCCCATATCACCAGCTCACCGTCTTTCATTGCCATACGGAATTCCGGATGAGCATTCATCAGGCGGGCAAGAATGTGAATAAAGGCCGGATAAAACTTGTGCTTATTTTTCTTTACGGTCTTTAAAAAGGCCGTAATATCCAGCTGAACGGTCTGGTTATAGGTACATTGAGCAACTGACTGAAATGCCTCAAAATGTTCTTTACGATGCCATTGGGATATATCAACGGTGGTATATCCAGTGATTTTTTTCTCCATTTTAGCTTCCTTAGCTCCTGAAAATCTCGATAACTCAAAAAATACGCCCGGTAGTGATCTTATTTCATTATGGTGAAAGTTGGAACCTCTTACGTGCCGATCAACGTCTCATTTTCGCCAAAAGTTGGCCCAGGGCTTCCCGGTATCAACAGGGACACCAGGATTTATTTATTCTGCGAAGTGATCTTCCGTCACAGGTATTTATTCGGCGCAAAGTGCGTCGGGTGATGCTGCCAACTTACTGATTTAGTGTATGATGGTGTTTTTGAGGTGCTCCAGTGGCTTCTGTTTCTATCAGCTGTCCCTCCTGTTCAGCTACTGACGGGGTGGTGCGTAACGGCAAAAGCACCGCCGGACATCAGCGCTTGTTTCGGCGTGGGTATGGTGGCAGGCCCCGTGGCCGGGGGACTGTTGGGCGCCTGTAGTGCCATTTACCCCCATTCACTGCCAGAGCCGTGAGCGCAGCGAACTGAATGTCACGAAAAAGACAGCGACTCAGGTGCCTGATGGTCGGAGACAAAAGGAATATTCAGCGATTTGCCCGAGCTTGCGAGGGTGCTACTTAAGCCTTTAGGGTTTTAAGGTCTGTTTTGTAGAGGAGCAAACAGCGTTTGCGACATCCTTTTGTAATACTGCGGAACTGACTAAAGTAGTGAGTTATACACAGGGCTGGGATCTATTCTTTTTATCTTTTTTTATTCTTTCTTTATTCTATAAATTATAACCACTTGAATATAAACAAAAAAAACACACAAAGGTCTAGCGGAATTTACAGAGGGTCTAGCAGAATTTACAAGTTTTCCAGCAAAGGTCTAGCAGAATTTACAGATACCCACAACTCAAAGGAAAAGGACTAGTAATTATCATTGACTAGCCCATCTCAATTGGTATAGTGATTAAAATCACCTAGACCAATTGAGATGTATGTCTGAATTAGTTGTTTTCAAAGCAAATGAACTAGCGATTAGTCGCTATGACTTAACGGAGCATGAAACCAAGCTAATTTTATGCTGTGTGGCACTACTCAACCCCACGATTGAAAACCCTACAAGGAAAGAACGGACGGTATCGTTCACTTATAACCAATACGTTCAGATGATGAACATCAGTAGGGAAAATGCTTATGGTGTATTAGCTAAAGCAACCAGAGAGCTGATGACGAGAACTGTGGAAATCAGGAATCCTTTGGTTAAAGGCTTTGAGATTTTCCAGTGGACAAACTATGCCAAGTTCTCAAGCGAAAAATTAGAATTAGTTTTTAGTGAAGAGATATTGCCTTATCTTTTCCAGTTAAAAAAATTCATAAAATATAATCTGGAACATGTTAAGTCTTTTGAAAACAAATACTCTATGAGGATTTATGAGTGGTTATTAAAAGAACTAACACAAAAGAAAACTCACAAGGCAAATATAGAGATTAGCCTTGATGAATTTAAGTTCATGTTAATGCTTGAAAATAACTACCATGAGTTTAAAAGGCTTAACCAATGGGTTTTGAAACCAATAAGTAAAGATTTAAACACTTACAGCAATATGAAATTGGTGGTTGATAAGCGAGGCCGCCCGACTGATACGTTGATTTTCCAAGTTGAACTAGATAGACAAATGGATCTCGTAACCGAACTTGAGAACAACCAGATAAAAATGAATGGTGACAAAATACCAACAACCATTACATCAGATTCCTACCTACATAACGGACTAAGAAAAACACTACACGATGCTTTAACTGCAAAAATTCAGCTCACCAGTTTTGAGGCAAAATTTTTGAGTGACATGCAAAGTAAGTATGATCTCAATGGTTCGTTCTCATGGCTCACGCAAAAACAACGAACCACACTAGAGAACATACTGGCTAAATACGGAAGGATCTGAGGTTCTTATGGCTCTTGTATCTATCAGTGAAGCATCAAGACTAACAAACAAAAGTAGAACAACTGTTCACCGTTACATATCAAAGGGAAAACTGTCCATATGCACAGATGAAAACGGTGTAAAAAAGATAGATACATCAGAGCTTTTACGAGTTTTTGGTGCATTTAAAGCTGTTCACCATGAACAGATCGACAATGTAACAGATGAACAGCATGTAACACCTAATAGAACAGGTGAAACCAGTAAAACAAAGCAACTAGAACATGAAATTGAACACCTGAGACAACTTGTTACAGCTCAACAGTCACACATAGACAGCCTGAAACAGGCGATGCTGCTTATCGAATCAAAGCTGCCGACAACACGGGAGCCAGTGACGCCTCCCGTGGGGAAAAAATCATGGCAATTCTGGAAGAAATAGCGCCTGTTTCGTTTCAGGCAGGTTATCAGGGAGTGTCAGCGTCCTGCGGTTCTCCGGGGCGTTCGGGTCATGCAGCCCGTAATGGTGATTTACCAGCGTCTGCCAGGCATCAATTCTAGGCCTGTCTGCGCGGTCGTAGTACGGCTGGAGGCGTTTTCCGGTCTGTAGCTCCATGTTCGGAATGACAAAATTCAGCTCAAGCCGTCCCTTGTCCTGGTGCTCCACCCACAGGATGCTGTACTGATTTTTTTCGAGACCGGGCATCAGTACACGCTCAAAGCTCGCCATCACTTTTTCACGTCCTCCCGGCGGCAGCTCCTTCTCCGCGAACGACAGAACACCGGACGTGTATTTCTTCGCAAATGGCGTGGCATCGATGAGTTCCCGGACTTCTTCCGGATTACCCTGAAGCACCGTTGCGCCTTCGCGGTTACGCTCCCTCCCCAGCAGGTAATCAACCGGACCACTGCCACCACCTTTTCCCCTGGCATGAAATTTAACTATCATCCCGCGCCCCCTGTTCCCTGACAGCCAGACGCAGCCGGCGCAGCTCATCCCCGATGGCCATCAGTGCGGCCACCACCTGAACCCGGTCACCGGAAGACCACTGCCCGCTGTTCACCTTACGGGCTGTCTGATTCAGGTTATTTCCGATGGCGGCCAGCTGACGCAGTAACGGCGGTGCCAGTGTCGGCAGTTTTCCGGAACGGGCAACCGGCTCCCCCAGGCAGACCCGCCGCATCCATACCGCCAGTTGTTTACCCTCACAGCGTTCAAGTAACCGGGCATGTTCATCATCAGTAACCCGTATTGTGAGCATCCTCTCGCGTTTCATCGGTATCATTACCCCATGAACAGAAATCCCCCTTACACGGAGGCATCAGTGACTAAACGGGGTCTGACGCTCAGTGGAACGAAAACTCACGTTAAGGGATTTTGGTCATGAGATTATCAAAAAGGATCTTCACCTAGATCCTTTTAAATTAAAAATGAAGTTTTAAATCAATCTAAAGTATATATGAGTAAACTTGGTCTGACAGTTACCAATGCTTAATCAGTGAGGCACCTATCTCAGCGATCTGTCTATTTCGTTCATCCATAGTTGCCTGACTCCCCGTCGTGTAGATAACTACGATACGGGAGGGCTTACCATCTGGCCCCAGTGCTGCAATGATACCGCGAGACCCACGCTCACCGGCTCCAGATTTATCAGCAATAAACCAGCCAGCCGGAAGGGCCGAGCGCAGAAGTGGTCCTGCAACTTTATCCGCCTCCATCCAGTCTATTAATTGTTGCCGGGAAGCTAGAGTAAGTAGTTCGCCAGTTAATAGTTTGCGCAACGTTGTTGCCATTGCTGCAGGCATCGTGGTGTCACGCTCGTCGTTTGGTATGGCTTCATTCAGCTCCGGTTCCCAACGATCAAGGCGAGTTACATGATCCCCCATGTTGTGCAAAAAAGCGGTTAGCTCCTTCGGTCCTCCGATCGTTGTCAGAAGTAAGTTGGCCGCAGTGTTATCACTCATGGTTATGGCAGCACTGCATAATTCTCTTACTGTCATGCCATCCGTAAGATGCTTTTCTGTGACTGGTGAGTACTCAACCAAGTCATTCTGAGAATAGTGTATGCGGCGACCGAGTTGCTCTTGCCCGGCGTCAACACGGGATAATACCGCGCCACATAGCAGAACTTTAAAAGTGCTCATCATTGGAAAACGTTCTTCGGGGCGAAAACTCTCAAGGATCTTACCGCTGTTGAGATCCAGTTCGATGTAACCCACTCGTGCACCCAACTGATCTTCAGCATCTTTTACTTTCACCAGCGTTTCTGGGTGAGCAAAAACAGGAAGGCAAAATGCCGCAAAAAAGGGAATAAGGGCGACACGGAAATGTTGAATACTCATACTCTTCCTTTTTCAATATTATTGAAGCATTTATCAGGGTTATTGTCTCATGAGCGGATACATATTTGAATGTATTTAGAAAAATAAACAAATAGGGGTTCCGCGCACATTTCCCCGAAAAGTGCCACCTGACGTCTAAGAAACCATTATTATCATGACATTAACCTATAAAAATAGGCGTATCACGAGGCCCTTTCGTCTTCAAGAATTTTATAAACCGTGGAGCGGGCAATACTGAGCTGATGAGCAATTTCCGTTGCACCAGTGCCCTTCTGATGAAGCGTCAGCACGACGTTCCTGTCCACGGTACGCCTGCGGCCAAATTTGATTCCTTTCAGCTTTGCTTCCTGTCGGCCCTCATTCGTGCGCTCTAGGATCCTCTACGCCGGACGCATCGTGGCCGGCATCACCGGCGCTGAGGTCTGCCTCGTGAAGAAGGTGTTGCTGACTCATACCAGGCCTGAATCGCCCCATCATCCAGCCAGAAAGTGAGGGAGCCACGGTTGATGAGAGCTTTGTTGTAGGTGGACCAGTTGGTGATTTTGAACTTTTGCTTTGCCACGGAACGGTCTGCGTTGTCGGGAAGATGCGTGATCTGATCCTTCAACTCAGCAAAAGTTCGATTTATTCAACAAAGCCGCCGTCCCGTCAAGTCAGCGTAATGCTCTGCCAGTGTTACAACCAATTAACCAATTCTGATTAGAAAAACTCATCGAGCATCAAATGAAACTGCAATTTATTCATATCAGGATTATCAATACCATATTTTTGAAAAAGCCGTTTCTGTAATGAAGGAGAAAACTCACCGAGGCAGTTCCATAGGATGGCAAGATCCTGGTATCGGTCTGCGATTCCGACTCGTCCAACATCAATACAACCTATTAATTTCCCCTCGTCAAAAATAAGGTTATCAAGTGAGAAATCACCATGAGTGACGACTGAATCCGGTGAGAATGGCAGAATAGGAACTTCGGAATAGGAACTTCAAAGCGTTTCCGAAAACGAGCGCTTCCGAAAATGCAACGCGAGCTGCGCACATACAGCTCACTGTTCACGTCGCACCTATATCTGCGTGTTGCCTGTATATATATATACATGAGAAGAACGGCATAGTGCGTGTTTATGCTTAAATGCGTACTTATATGCGTCTATTTATGTAGGATGAAAGGTAGTCTAGTACCTCCTGTGATATTATCCCATTCCATGCGGGGTATCGTATGCTTCCTTCAGCACTACCCTTTAGCTGTTCTATATGCTGCCACTCCTCAATTGGATTAGTCTCATCCTTCAATGCTATCATTTCCTTTGATATTGGATCATATGCATAGTACCGAGAAACTAGTGCGAAGTAGTGATCAGGTATTGCTGTTATCTGATGAGTATACGTTGTCCTGGCCACGGCAGAAGCACGCTTATCGCTCCAATTTCCCACAACATTAGTCAACTCCGTTAGGCCCTTCATTGAAAGAAATGAGGTCATCAAATGTCTTCCAATGTGAGATTTTGGGCCATTTTTTATAGCAAAGATTGAATAAGGCGCATTTTTCTTCAAAGCTTTATTGTACGATCTGACTAAGTTATCTTTTAATAATTGGTATTCCTGTTTATTGCTTGAAGAATTGCCGGTCCTATTTACTCGTTTTAGGACTGGTTCAGAATTCCTCAAAAATTCATCCAAATATACAAGTGGATCGATCCTACCCCTTGCGCTAAAGAAGTATATGTGCCTACTAACGCTTGTCTTTGTCTCTGTCACTAAACACTGGATTATTACTCCCAGATACTTATTTTGGACTAATTTAAATGATTTCGGATCAACGTTCTTAATATCGCTGAATCTTCCACAATTGATGAAAGTAGCTAGGAAGAGGAATTGGTATAAAGTTTTTGTTTTTGTAAATCTCGAAGTATACTCAAACGAATTTAGTATTTTCTCAGTGATCTCCCAGATGCTTTCACCCTCACTTAGAAGTGCTTTAAGCATTTTTTTACTGTGGCTATTTCCCTTATCTGCTTCTTCCGATGATTCGAACTGTAATTGCAAACTACTTACAATATCAGTGATATCAGATTGATGTTTTTGTCCATAGTAAGGAATAATTGTAAATTCCCAAGCAGGAATCAATTTCTTTAATGAGGCTTCCAGAATTGTTGCTTTTTGCGTCTTGTATTTAAACTGGAGTGATTTATTGACAATATCGAAACTCAGCGAATTGCTTATGATAGTATTATAGCTCATGAATGTGGCTCTCTTGATTGCTGTTCCGTTATGTGTAATCATCCAACATAAATAGGTTAGTTCAGCAGCACATAATGCTATTTTCTCACCTGAAGGTCTTTCAAACCTTTCCACAAACTGACGAACAAGCACCTTAGGTGGTGTTTTACATAATATATCAAATTGTGGCATACAACCTCCTTAGTACATGCAACCATTATCACCGCCAGAGGTAAAATAGTCAACACGCACGGTGTTAGATATTTATCCCTTGCGGTGATAGATTTAACGTATGAGCACAAAAAAGAAACCATTAACACAAGAGCAGCTTGAGGACGCACGTCGCCTTAAAGCAATTTATGAAAAAAAGAAAAATGAACTTGGCTTATCCCAGGAATCTGTCGCAGACAAGATGGGGATGGGGCAGTCAGGCGTTGGTGCTTTATTTAATGGCATCAATGCATTAAATGCTTATAACGCCGCATTGCTTACAAAAATTCTCAAAGTTAGCGTTGAAGAATTTAGCCCTTCAATCGCCAGAGAAATCTACGAGATGTATGAAGCGGTTAGTATGCAGCCGTCACTTAGAAGTGAGTATGAGTACCCTGTTTTTTCTCATGTTCAGGCAGGGATGTTCTCACCTAAGCTTAGAACCTTTACCAAAGGTGATGCGGAGAGATGGGTAAGCACAACCAAAAAAGCCAGTGATTCTGCATTCTGGCTTGAGGTTGAAGGTAATTCCATGACCGCACCAACAGGCTCCAAGCCAAGCTTTCCTGACGGAATGTTAATTCTCGTTGACCCTGAGCAGGCTGTTGAGCCAGGTGATTTCTGCATAGCCAGACTTGGGGGTGATGAGTTTACCTTCAAGAAACTGATCAGGGATAGCGGTCAGGTGTTTTTACAACCACTAAACCCACAGTACCCAATGATCCCATGCAATGAGAGTTGTTCCGTTGTGGGGAAAGTTATCGCTAGTCAGTGGCCTGAAGAGACGTTTGGCTGATCGGCAAGGTGTTCTGGTCGGCGCATAGCTGATAACAATTGAGCAAGAATCTGCATTTCTTTCCAGACTTGTTCAACAGGCCAGCCATTACGCTCGTCATCAAAATCACTCGCATCAACCAAACCGTTATTCATTCGTGATTGCGCCTGAGCGAGACGAAATACGCGATCGCTGTTAAAAGGACAATTACAAACAGGAATCGAATGCAACCGGCGCAGGAACACTGCCAGCGCATCAACAATATTTTCACCTGAATCAGGATATTCTTCTAATACCTGGAATGCTGTTTTCCCGGGGATCGCAGTGGTGAGTAACCATGCATCATCAGGAGTACGGATAAAATGCTTGATGGTCGGAAGAGGCATAAATTCCGTCAGCCAGTTTAGTCTGACCATCTCATCTGTAACATCATTGGCAACGCTACCTTTGCCATGTTTCAGAAACAACTCTGGCGCATCGGGCTTCCCATACAATCGATAGATTGTCGCACCTGATTGCCCGACATTATCGCGAGCCCATTTATACCCATATAAATCAGCATCCATGTTGGAATTTAATCGCGGCCTCGAGCAAGACGTTTCCCGTTGAATATGGCTCATAACACCCCTTGTATTACTGTTTATGTAAGCAGACAGTTTTATTGTTCATGATGATATATTTTTATCTTGTGCAATGTAACATCAGAGATTTT |

**Supplementary Table 3** Primers used for genome integration

| Primer name | Sequence |
| --- | --- |
| PKD3-malEK insert Cm F | GACCTTTGAAGGTCGGCAGTACCGTTACACCATAATTCACTTTACTGGTGTGTAGGCTGGAGCTGCTTC |
| PKD3-malEK insert Cm R | CCATCTGGTGGCGGGTTTAACGTCCTCCTTAGTTCCTATTCC |
| pACYC-malEK:: pACYC-araBAD -*tdc(R)* F | GGAATAGGAACTAAGGAGGACGTTAAACCCGCCACCAGATGG |
| pACYC-malEK:: pACYC-araBAD -*tdc(R)* R | CGACGACCTGAACTTCACCCTCAAGGATGACGTCAGCGATATCACTCGGCTGCTGCAATGATACCGCGAGAC |
| EcN malEK :: pACYC-araBAD-*tdc(R)* check F | TTCGGACTGGCGGCGTTAATAC |
| EcN malEK :: pACYC-araBAD-*tdc(R)* check R | CTACCAACACCACGTCGTTCTG |

**Supplementary Table 4** Primers used in Real-Time PCR assay.

| Primer name | Sequence |
| --- | --- |
| 5-HTR4-F(Engevik et al., 2021) | TCGGCATAGTTGATGTGATAGAGAAA |
| 5-HTR4-R(Engevik et al., 2021) | ATGAGGAGAAACGGGATGTAGAAGG |
| β-actin(F) | TGGATGACGATATCGCTGCG |
| β-actin(R) | AGGGTCAGGATACCTCTCTT |
| EcN pMUT2 qPCR-F (Blum-Oehler et al., 2003) | GACCAAGCGATAACCGGATG |
| EcN pMUT2 qPCR-R (Blum-Oehler et al., 2003) | GTGAGATGATGGCCACGATT |

**Reference**

BLUM-OEHLER, G., OSWALD, S., EITELJöRGE, K., SONNENBORN, U., SCHULZE, J., KRUIS, W. & HACKER, J. 2003. Development of strain-specific PCR reactions for the detection of the probiotic Escherichia coli strain Nissle 1917 in fecal samples. *Research in microbiology,* 154**,** 59-66. doi: 10.1016/S0923-2508(02)00007-4

ENGEVIK, M. A., LUCK, B., VISUTHRANUKUL, C., IHEKWEAZU, F. D., ENGEVIK, A. C., SHI, Z., DANHOF, H. A., CHANG-GRAHAM, A. L., HALL, A., ENDRES, B. T., HAIDACHER, S. J., HORVATH, T. D., HAAG, A. M., DEVARAJ, S., GAREY, K. W., BRITTON, R. A., HYSER, J. M., SHROYER, N. F. & VERSALOVIC, J. 2021. Human-Derived Bifidobacterium dentium Modulates the Mammalian Serotonergic System and Gut-Brain Axis. *Cell Mol Gastroenterol Hepatol,* 11**,** 221-248. doi: 10.1016/j.jcmgh.2020.08.002
